# Culture impact on the transcriptomic programs of primary and iPSC-derived human alveolar type 2 cells

**DOI:** 10.1172/jci.insight.158937

**Published:** 2023-01-10

**Authors:** Konstantinos-Dionysios Alysandratos, Carolina Garcia-de-Alba, Changfu Yao, Patrizia Pessina, Jessie Huang, Carlos Villacorta-Martin, Olivia T. Hix, Kasey Minakin, Claire L. Burgess, Pushpinder Bawa, Aditi Murthy, Bindu Konda, Michael F. Beers, Barry R. Stripp, Carla F. Kim, Darrell N. Kotton

**Affiliations:** 1 Center for Regenerative Medicine, Boston University and Boston Medical Center, Boston, Massachusetts, USA.; 2 The Pulmonary Center and Department of Medicine, Boston University Chobanian & Avedisian School of Medicine, Boston, Massachusetts, USA.; 3 Stem Cell Program and Divisions of Hematology/Oncology and Pulmonary Medicine, Boston Children’s Hospital, Boston, Massachusetts, USA.; 4 Harvard Stem Cell Institute, Cambridge, Massachusetts, USA.; 5 Department of Genetics, Harvard Medical School, Boston, Massachusetts, USA.; 6 Women’s Guild Lung Institute,; 7 Division of Pulmonary and Critical Care Medicine, Department of Medicine, and; 8 Regenerative Medicine Institute, Cedars-Sinai Medical Center, Los Angeles, California, USA.; 9 Stem Cells and Regenerative Medicine Center, Baylor College of Medicine, Houston, Texas, USA.; 10 Pulmonary, Allergy, and Critical Care Division, Department of Medicine, and; 11 PENN-CHOP Lung Biology Institute, University of Pennsylvania Perelman School of Medicine, Philadelphia, Pennsylvania, USA.

**Keywords:** Pulmonology, Stem cells, Human stem cells, Molecular biology, iPS cells

## Abstract

Dysfunction of alveolar epithelial type 2 cells (AEC2s), the facultative progenitors of lung alveoli, is implicated in pulmonary disease pathogenesis, highlighting the importance of human in vitro models. However, AEC2-like cells in culture have yet to be directly compared to their in vivo counterparts at single-cell resolution. Here, we performed head-to-head comparisons among the transcriptomes of primary (1°) adult human AEC2s, their cultured progeny, and human induced pluripotent stem cell–derived AEC2s (iAEC2s). We found each population occupied a distinct transcriptomic space with cultured AEC2s (1° and iAEC2s) exhibiting similarities to and differences from freshly purified 1° cells. Across each cell type, we found an inverse relationship between proliferative and maturation states, with preculture 1° AEC2s being most quiescent/mature and iAEC2s being most proliferative/least mature. Cultures of either type of human AEC2s did not generate detectable alveolar type 1 cells in these defined conditions; however, a subset of iAEC2s cocultured with fibroblasts acquired a transitional cell state described in mice and humans to arise during fibrosis or following injury. Hence, we provide direct comparisons of the transcriptomic programs of 1° and engineered AEC2s, 2 in vitro models that can be harnessed to study human lung health and disease.

## Graphical abstract

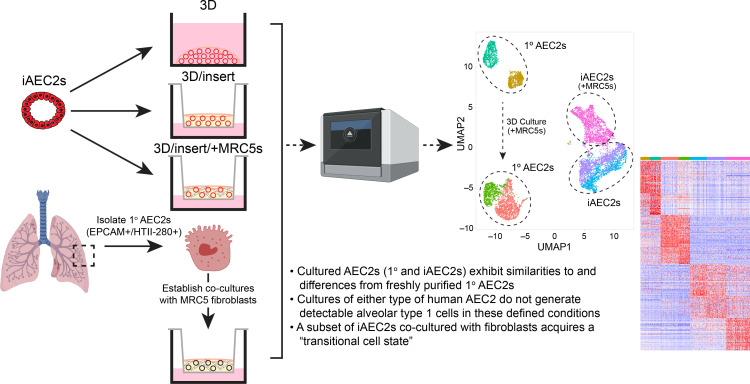

## Introduction

Lung alveolar epithelial type 2 cells (AEC2s) fulfill specialized functions in their quiescent state and serve as facultative progenitors, able to reenter the cell cycle to maintain the alveolar epithelium after injury. Dysfunction of this key cell type has been implicated in the pathogenesis of a number of pulmonary diseases, including pulmonary fibrosis (1–4). Limited access to primary (1°) AEC2s from patients and difficulties with their phenotypic maintenance in long term ex vivo cultures have impeded development of tractable in vitro disease models. Prior investigations have focused on the development of methods for purification and in vitro propagation of rodent or human AEC2s, initially using 2D culture methods (5–8) and more recently using 3D culture conditions with (9–12) or without (13–16) supporting feeder cells. Early attempts to maintain AEC2s in 2D culture were limited by rapid loss of AEC2-specific gene expression and loss of proliferative capacity (8, 17), with several investigators noting upregulation of gene markers, interpreted by some (5, 17–19), but not all (20), as indicating differentiation of cuboidal AEC2s into squamous appearing alveolar epithelial type 1–like (AEC1-like) cells.

With the advent of 3D culture models, we and others have developed methods for the in vitro culture of human AEC2-like cells derived from 1° fetal (7), 1° adult (10, 14–16, 21, 22), or induced pluripotent stem cell (iPSC) sources (13, 22, 23). These methods have enabled the in vitro maintenance of functional human AEC2-like cells that share similar transcriptional and ultrastructural properties to their in vivo or freshly isolated 1° AEC2 counterparts, including the capacity to produce surfactant proteins and phospholipids (13–16, 23). Despite these advances, which enable more prolonged maintenance in cell culture of human AEC2-like cells, many controversies and questions remain to be addressed. For example, 1° or iPSC-derived AEC2-like cells captured in cell culture have been compared with in vivo 1° cells by bulk methods, such as reverse transcription quantitative polymerase chain reaction (RT-qPCR) (23) or RNA sequencing (RNA-Seq) (13, 23), but have yet to be directly compared head-to-head (without potential technical batch effects) at the single-cell level with their uncultured 1° cell counterparts. This raises uncertainty regarding how closely either 1° derived or iPSC-derived AEC2-like cells resemble in vivo AEC2 controls, in terms of global gene expression profiles, heterogeneity, and proliferation states. In addition, the differentiation repertoire of human AEC2s either in vivo or in vitro remains uncertain. Few studies have provided comprehensive profiles, beyond just a few selected markers of unclear specificity, of human AEC1s arising from human AEC2s in culture (reviewed in ref. 22). Furthermore, some studies (10) found no evidence of AEC1s arising from 1° adult human AEC2s when cultured as alveolospheres. These findings for cultured human AEC2s are in contrast with observations made in rodents, where lineage-tracing studies as well as single-cell RNA-Seq (scRNA-Seq) profiles have established that adult rat or mouse AEC2s give rise to AEC1s both in vitro and in vivo (10, 24–27). In addition, rapidly emerging literature has revealed a variety of transitional lung epithelial states (also referred to in the literature as intermediate cell states, aberrant basaloid cells, damage-associated transient progenitors [DATPs], *KRT5^–^KRT17^+^* cells, pre-alveolar type 1 transitional cell state [PATS], Krt8^+^ alveolar differentiation intermediate [ADI] state, or alveolar-basal intermediates [ABIs]) appearing in cultured mouse AEC2 samples, in mouse lung injury models, or in the distal lung tissues of patients with fibrosing illnesses (24, 25, 27–30). These transitional cells have been identified by a diversity of markers not expressed in normal AEC2s, such as basal cell–like cytokeratins. Although neither the pathogenic/reparative potential of these transitional cells nor their cellular origins have been clearly determined, some have proposed AEC2s as their source (24, 25, 27, 31, 32); thus, whether analogous cells can be generated for study in vitro from human AEC2s is of increasing interest to those studying pulmonary fibrosis.

Here, we performed head-to-head comparisons between the single-cell transcriptomes of 1° adult human AEC2s (prior to culturing), their isogenic cultured progeny, and human iPSC-derived AEC2s (iAEC2s) cultured in the identical defined medium with and without feeders. We found that each population (1° preculture, 1° cultured, and iPSC-derived) expressed a distinct transcriptomic profile. Both cultured cell populations exhibited maintenance of the AEC2-like phenotype in vitro, with gene expression similarities with freshly captured adult 1° AEC2s. We provided quantitative correlation scores comparing each 1° and cultured AEC2 population, and we observed a gradient of cell cycle states and maturation gene expression levels that distinguished each cell preparation. Neither population of cultured AEC2s showed evidence of differentiation toward bona fide AEC1s when cultured in these conditions, but a subset of iAEC2s when cocultured with mesenchymal cells upregulated markers of transitional cell phenotypes reminiscent of the recently described transitional cell states observed in mice (24, 25, 31, 33) and humans (25, 31, 34) during alveolar repair after lung injury and overrepresented in human lung diseases such as idiopathic pulmonary fibrosis (IPF) (25, 28–31).

## Results

### Establishment of synchronous 1° AEC2 and iAEC2 cultures.

**Figure 1:**
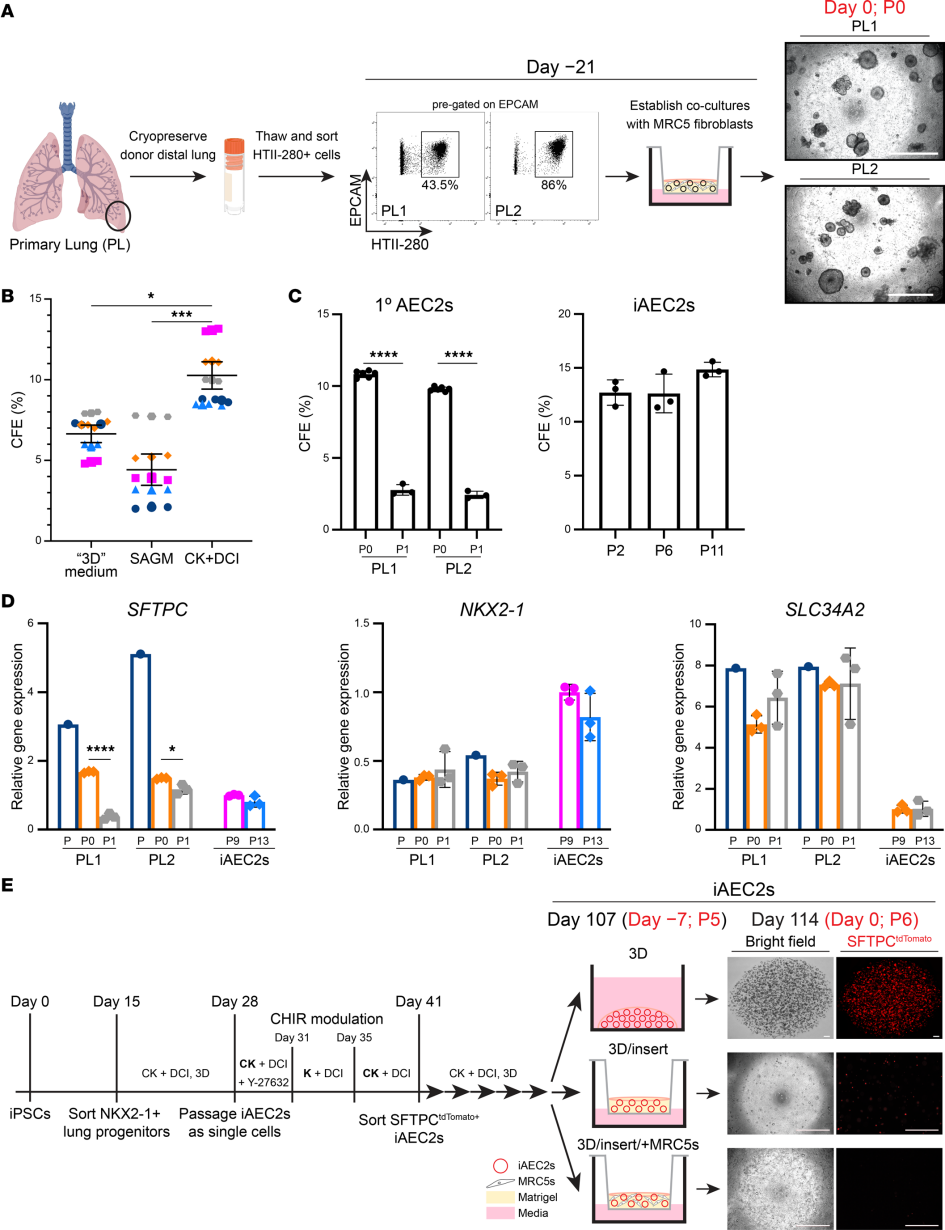
Establishment of synchronous 1° AEC2 and iAEC2 cultures. (**A**) Schematic depicting the cryopreservation of distal lung preparations from adult donor lung explants (PL1, 2) and FACS gates used to isolate 1° AEC2s (EPCAM^+^HTII-280^+^ cells), which were combined with MRC5 fibroblasts on cell culture inserts on day –21. Representative live-cell imaging of the outgrowths on the day of encapsulation for scRNA-Seq (day 0). (**B**) Super plot shows the colony-forming efficiency (CFE) of 1° AEC2s in 3 media. Small shapes represent replicate values (*n* = 3) from each independent donor, and color-matched large shapes represent the average for each donor (*n* = 5). (**C**) Bar graphs showing CFE after the first plating in culture prior to passaging (P0), reduced CFE of passaged (P1) 1° AEC2s (*n* = 2 donors), and stable CFE of iAEC2s across multiple passages (*n* = 3 experimental replicates). No colonies were formed from P2 1° AEC2s. (**D**) RT-qPCR showing fold change in gene expression compared with P9 iAEC2s in P13 iAEC2s and preculture (P) and cultured (P0 and P1) 1° AEC2s from 2 donors (PL1 and PL2) (*n* = 3 experimental replicates). (**E**) Schematic of directed differentiation protocol from iPSCs to day 107 (P5) iAEC2s. Seven days prior to encapsulation for scRNA-Seq (day –7), 3D iAEC2s were dissociated and plated in 3 parallel conditions: 1) continued 3D feeder-free iAEC2 cultures, 2) 3D feeder-free cultures on cell culture inserts (3D/insert), or 3) 3D cultures on cell culture inserts with MRC5 fibroblasts (3D/insert/+MRC5s) identical to conditions for the 1° AEC2s. Representative live-cell imaging of the outgrowths on day 0. (**A** and **E**) Scale bars: 500 μm. Mean ± SEM (**B**) and mean ± SD (**C** and **D**) shown; *P < 0.05, ***P < 0.001, ****P < 0.0001 by 1-way ANOVA with Tukey’s correction for multiple comparisons (**B**) or unpaired, 2-tailed Student’s *t* test (**C** and **D**).

To perform head-to-head comparisons between the global transcriptomes of 1° AEC2s, cultured 1° AEC2s, and cultured iAEC2s, we sought to establish synchronous cultures with similar conditions that would allow control of potential batch and media effects. Distal lung preparations from adult donor lung explants from 5 individuals (primary lung 1–5, PL 1–5) were cryopreserved using methods we recently described (ref. 21 and depicted in Figure 1A). After thawing, AEC2s were purified using FACS to isolate cells coexpressing EPCAM and the AEC2-selective surface marker HTII-280 (35). Once sorted, these cells were combined with MRC5 fibroblasts on cell culture inserts, and in vitro colony-forming efficiency (CFE) was scored after outgrowth in 3 media: CK+DCI, a defined serum-free medium containing CHIR99021, keratinocyte growth factor (KGF), dexamethasone, cyclic AMP, and 3-Isobutyl-1-methylxanthine (IBMX) that we have previously published for maintenance of human iAEC2s (13); 3D medium (10% FBS–containing medium) previously published for the coculture of mouse AEC2s with mesenchymal cells (11); or small airway epithelial cell growth medium (SAGM) (12) (Figure 1, A and B). CK+DCI medium resulted in significantly higher CFE than the other 2 media (Figure 1B) and was thus chosen for further studies comparing cultured 1° AEC2s versus iAEC2s in identical medium. Primary AEC2s cultured in the absence of supporting MRC5 fibroblasts did not yield any outgrowth colonies in these media (data not shown), consistent with our prior description of the need for supporting cells in order to expand 1° AEC2s in culture (13). To test the expansion potential of 1° AEC2s cocultured with MRC5 fibroblasts, we monitored growth kinetics and expression of the AEC2 program after serial passaging of each sample. After 1 passage (P1), 1° cultured AEC2s demonstrated significantly reduced CFE compared with the starting passage (P0) (Figure 1C) and displayed reduced expression of some AEC2-specific transcripts (e.g., *SFTPC*), but maintenance of others (e.g., *NKX2-1* and *SLC34A2*), when compared with preculture and P0 1° AEC2s (Figure 1D). Primary AEC2s could not be propagated beyond 2 serial passages (Figure 1C), whereas iAEC2s could be passaged indefinitely with no requirement for fibroblasts (Figure 1C), as previously published (36, 37). iAEC2s expressed *SFTPC* at similar levels, *NKX2-1* at higher levels, and *SLC34A2* at lower levels compared with 1° P1 AEC2s (Figure 1D). Using samples from an additional donor (PL5), we validated the findings of reduced expression of the AEC2-specific transcript *SFTPC* and showed increased expression of the proliferation-related transcript *MKI67* in cultured 1° AEC2s (P0) compared with their preculture counterparts (Supplemental Figure 1A; supplemental material available online with this article; https://doi.org/10.1172/jci.insight.158937DS1), suggesting that cultured AEC2s acquired a less mature and more proliferative state. Notably, there were no significant differences in CFE or ability to be passaged related to donor age, though the relatively small sample size in this study precludes definitive conclusions related to impact of donor age on culture performance.

To establish synchronous cultures of 1° AEC2s and iAEC2s for profiling of single-cell transcriptomes, we used our previously published lung directed differentiation protocol (13, 38) to establish 3D cultures of indefinitely self-renewing pure iAEC2s from a human iPSC line carrying a tdTomato fluorescent reporter targeted to the *SFTPC* locus (SPC2 line; SPC2-ST-B2 clone) (Figure 1E) (36, 37). In these feeder-free 3D culture conditions, iAEC2s demonstrated maintenance of an AEC2-specific transcriptomic profile (Figure 1D; see also ref. 37). In parallel wells, we dissociated 3D iAEC2s and plated their progeny in CK+DCI medium in 3 parallel conditions — i) continued 3D feeder-free iAEC2 cultures, ii) 3D feeder-free cultures on cell culture inserts (3D/insert), or iii) 3D cultures on cell culture inserts with MRC5 fibroblasts (3D/insert/+MRC5s) (Figure 1E) — that were identical to conditions for the 1° AEC2s, cultured in parallel. In contrast to iAEC2s maintained in 3D feeder-free conditions, coculture of iAEC2s with MRC5 fibroblasts resulted in loss of SFTPC^tdTomato^ expression (Figure 1E), suggesting that these fibroblasts alter the molecular phenotype of iAEC2s.

### Single-cell transcriptomic profiling of freshly isolated 1° AEC2s, cultured 1° AEC2s, and iAEC2s.

**Figure 2:**
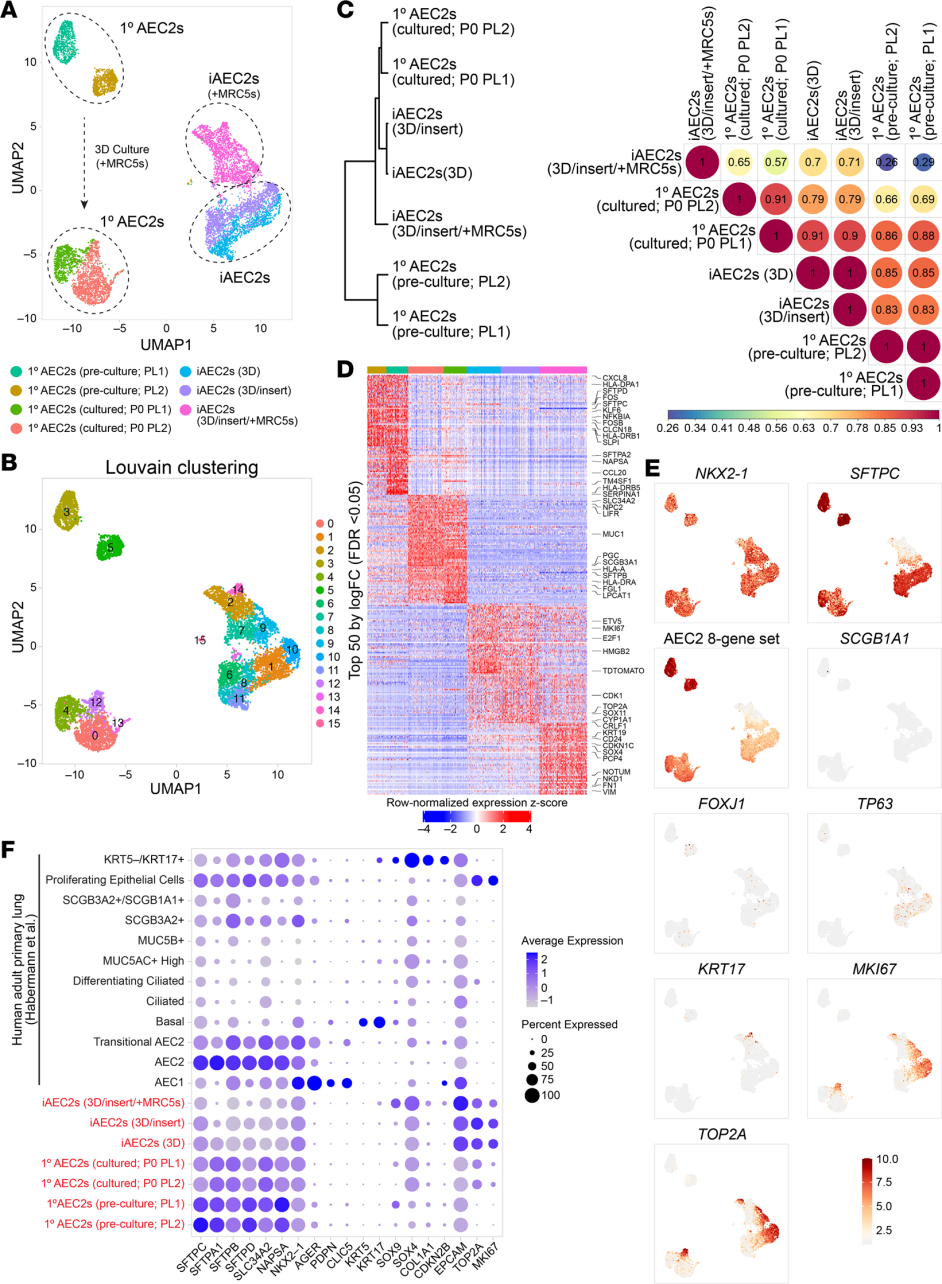
Single-cell transcriptomic profiling of 1° AEC2s and iAEC2s. (**A**) Visualization of preculture 1° AEC2, cultured 1° AEC2, and iAEC2 scRNA-Seq transcriptomes using uniform manifold approximation projection (UMAP). (**B**) Louvain clustering of cell transcriptomes identifies 16 different clusters driven primarily by sample type or donor identity, but with subclustering within cultured cell populations suggesting the presence of cell heterogeneity in vitro. (**C**) Dendrogram and heatmap of Pearson’s correlation coefficients between each sample based on normalized expression of the 3,000 most variable genes across all cells. (**D**) Heatmap of top 50 differentially upregulated genes for each sample by scRNA-Seq (ranked by average log fold change, FDR < 0.05; row-normalized expression *z* scores). A subset of differentially expressed genes is highlighted with large font. (**E**) Normalized gene expression overlaid on UMAP plots for the indicated transcripts or gene sets. (**F**) Average expression levels and frequencies (purple dots) for select genes profiled by scRNA-Seq in preculture 1° AEC2s, cultured 1° AEC2s, and iAEC2s. Comparison is made to a publicly available adult 1° distal lung data set (30), and genes are selected to indicate AEC2, AEC1, airway, epithelial, or proliferation programs.

Next, we used scRNA-Seq to compare 7 samples, all prepared in parallel and sequenced on the same day to avoid technical batch effects. We profiled each of the 3 iAEC2 preparations and four 1° AEC2 samples consisting of (a) preculture 1° AEC2s, defined as freshly isolated, cryopreserved HTII-280^+^ 1° AEC2s FACS-purified from the same 2 donor lungs used to establish the 1° AEC2 cultures (Figure 1A), and (b) cultured 1° AEC2s, defined as the P0 1° AEC2 progeny of the preculture 1° AEC2s after 21 days of culture with MRC5 fibroblasts in CK+DCI medium on cell culture inserts. The iAEC2 sample cocultured with MRC5s on inserts and the 2 cultured 1° AEC2 samples were sorted for viable EPCAM^+^ cells before scRNA-Seq. All other samples were sorted for live cells only. To harvest cells of similar confluence despite different growth kinetics of 1° AEC2s versus iAEC2s, iAEC2s were harvested on day 7 after passage, whereas P0 1° AEC2s were harvested on day 21. The transcriptomes of each AEC2 population occupied distinct transcriptomic spaces when visualized by uniform manifold approximation and projection (UMAP; Figure 2A). Louvain clustering identified 16 clusters (Figure 2B) driven primarily by sample type or donor identity but with subclustering within cultured cell populations suggesting the presence of cell heterogeneity in vitro.

To quantitatively score similarities in gene expression between each sample, we plotted the normalized expression of the 3,000 most variable genes across all cells and calculated Pearson’s correlation coefficients for pairwise sample comparisons (Figure 2C). We observed high gene expression correlations between each sample with the exception of MRC5-cocultured iAEC2s. Specifically, preculture versus cultured 1° AEC2s displayed high correlations, as did feeder-free iAEC2s versus preculture 1° AEC2s, and feeder-free iAEC2 versus cultured 1° AEC2s. In contrast, iAEC2s after coculturing with MRC5s displayed lower correlations with all other samples (Figure 2C). Together, these data indicate that cultured 1° AEC2s and feeder-free iAEC2s exhibit transcriptomic similarities to and differences from preculture 1° AEC2s.

Focusing first on the canonical AEC2 marker, *SFTPC*, as anticipated by bulk RT-qPCR profiles (Figure 1D and Supplemental Figure 1), we observed significantly higher expression of this transcript in preculture cells by scRNA-Seq, though expression was easily detected in the vast majority of cultured 1° AEC2s or iAEC2s (Figure 2, D–F). In contrast, iAEC2s cocultured with MRC5 fibroblasts expressed less *SFTPC* (Figure 2, D and E), as predicted, based on loss of SFTPC^tdTomato^ reporter expression (Figure 1E) and lower *NKX2-1* expression (Figure 2D). This loss of *SFTPC* in cocultured iAEC2s was consistent with their lower Pearson’s correlation scores, compared with other cells (Figure 2C). In addition to loss of *SFTPC* in this sample, 2 distinct cell clusters (cluster 14 and related cluster 2; Figure 2B) enriched in cytokeratins (*KRT8*, *KRT17*, *KRT19*) emerged (Figure 2, D–F, and Supplemental Figure 2, and further discussed below). Importantly, cultured 1° or iPSC-derived cells did not detectably assume alternative lung fates based on little to no expression of airway (*SCGB1A1*, *FOXJ1*, *TP63*, *KRT5*; Figure 2, E and F) or AEC1 transcripts (Figure 2F).

Next, we assessed the most significant differences in gene expression between samples. Among the top 50 differentially upregulated genes in preculture 1° AEC2s compared with all other cells (Figure 2D) were transcripts associated with AEC2 differentiation or maturation (36) (*SFTPA2*, *SFTPC*, *SFTPD*, *NAPSA*, *SLPI*) and immune-related transcripts (*CXCL1*, *CXCL2*, *CXCL3*, *CXCL8*, *CCL2*, *CCL20*, *HLA-DPA1*, *HLA-DRB1*, *HLA-DRB5*, *NFKBIA*, *NFKBIZ*, *TNFRSF12A*, *TNFAIP3*, *CD83*). Cultured 1° AEC2s were also enriched in transcripts encoding AEC2 marker genes, although their expression levels were lower than their preculture counterparts. iAEC2s were more proliferative than cultured 1° AEC2s based on significantly higher expression of *MKI67*, *TOP2A*, and transcripts associated with cytokinesis (Figure 2, D–F). iAEC2s expressed the canonical AEC2 marker transcript *SFTPC* and the transcript for the SFTPC^tdTomato^ reporter (Figure 2, D–F). Compared with all other cells, iAEC2s cultured in feeder-free conditions expressed significantly higher levels of some distal alveolar epithelial marker transcripts (*ETV5*, *CRLF1*; Figure 2D) and lower levels of most mature AEC2 marker transcripts (*SFTPA2*, *SFTPD*, *NAPSA*, *SLPI*). At the protein level, preculture 1° AEC2s and cultured AEC2s (P0 1° AEC2s and iAEC2s) expressed and fully processed SFTPB protein to the mature 8 kDa isoform as well as SFTPC protein to the mature 3.7 kDa form (Supplemental Figure 1B; see complete unedited blots in the supplemental material), indicating the presence of functional lamellar bodies across all samples. Levels of processed SFTPB protein were lower in iAEC2s compared with primary cells, concordant with *SFTPB* mRNA expression levels (Figure 2). The processing of both SFTPB and SFTPC to their mature forms in iAEC2s is consistent with prior reports examining surfactant protein processing in iAEC2s generated from a variety of human iPSCs or embryonic stem cells of various genetic backgrounds (13, 37, 39).

**Figure 3:**
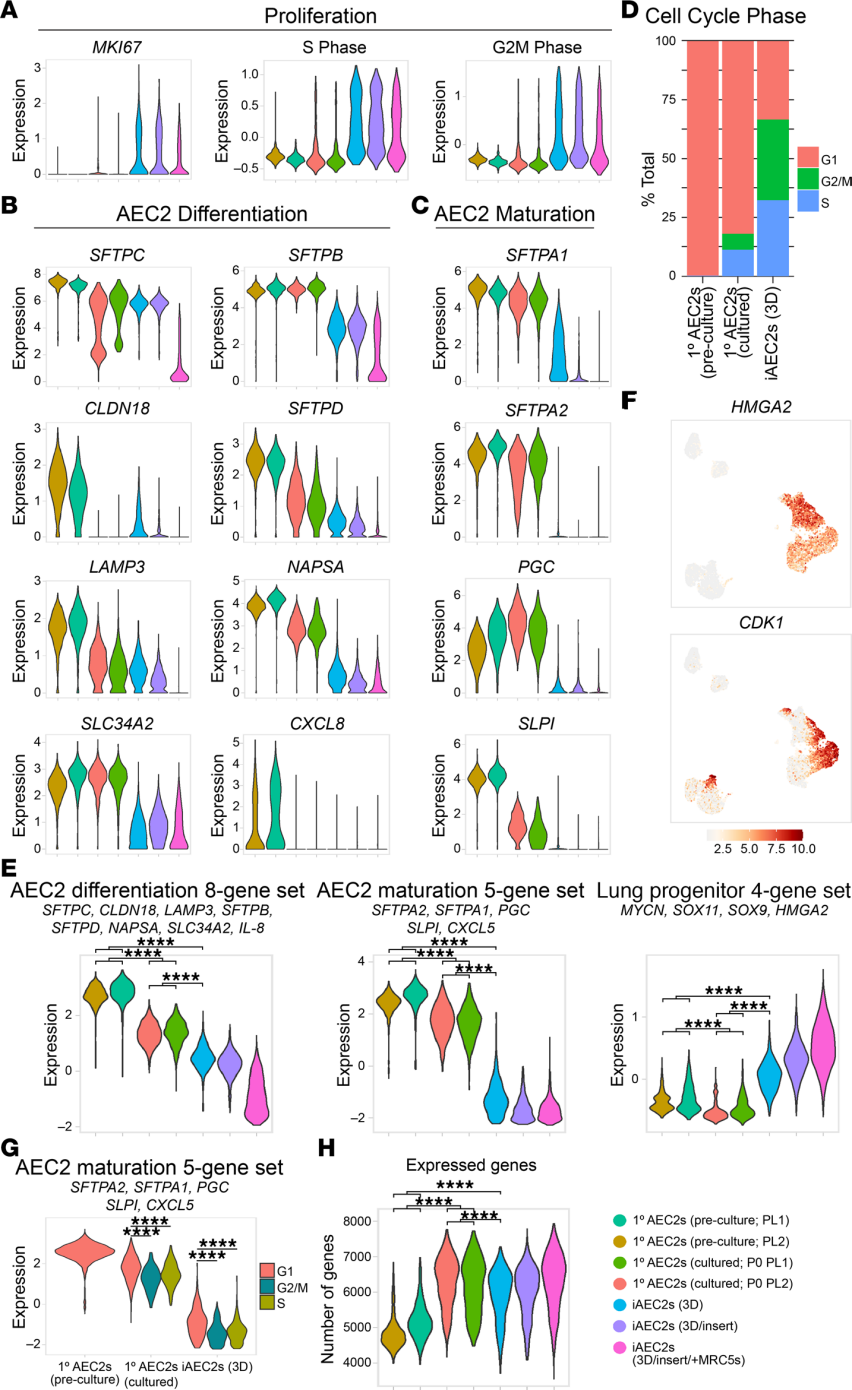
AEC2 maturation is inversely related to proliferation. (**A**) Violin plots showing normalized expression for *MKI67* and cell cycle phase in preculture 1° AEC2s, cultured 1° AEC2s, and iAEC2s by scRNA-Seq. (**B**) Violin plots showing normalized expression for individual genes from a published AEC2 differentiation gene set (36). (**C**) Violin plots showing normalized expression for individual genes from a published maturation gene set (36). (**D**) Bar plot of cell cycle phase proportions by sample. (**E**) Violin plots showing normalized expression for indicated gene sets in preculture 1° AEC2s, cultured 1° AEC2s, and iAEC2s by scRNA-Seq. (**F**) Normalized gene expression overlaid on UMAP plots for the indicated transcripts. (**G**) Violin plots of AEC2 maturation gene set by cell cycle phase in preculture 1° AEC2s, cultured 1° AEC2s, and iAEC2s. (**H**) Violin plots of expressed genes in preculture 1° AEC2s, cultured 1° AEC2s, and iAEC2s by scRNA-Seq. ****P < 0.0001 by 1-way ANOVA with Bonferroni correction for multiple comparisons for all panels.

Because prior studies have observed an inverse relationship between the proliferation and maturation programs in AEC2s (13, 39, 40), we next focused on comparing the proliferation and maturation transcriptomic programs across all 7 samples. We found a continuum of progressively more proliferative states (from preculture 1° to cultured 1° to iAEC2s) that were inversely associated with gene signatures of AEC2 maturation (Figure 3, A–C). Specifically, scRNA-Seq data demonstrated that cultured AEC2s (1° or iAEC2s) were more proliferative compared with preculture 1° AEC2s, as expected. While 66.5% of iAEC2s and 18% of cultured 1° AEC2s expressed transcripts associated with active cell cycling (S, G2, or M phase), only 0.1% of preculture 1° AEC2s expressed such transcripts (Figure 3D). In contrast, top genes upregulated in preculture 1° AEC2s included transcripts encoding surfactants and lamellar body–related and other AEC2 marker genes (Figure 2, B, C, and E). Similarly, preculture 1° AEC2s expressed higher levels of genes and gene sets (36) associated with AEC2 differentiation (Figure 3, B and E) and maturation (Figure 3, C and E) whereas cultured AEC2s expressed higher levels of genes (*HMGA2*, *CDK1*) and gene sets (*MYCN*, *SOX11*, *SOX9*, *HMGA2*; ref. 36) associated with progenitor cells (Figure 3, E and F). Across the different samples, cells that were not in S/G2/M phases expressed significantly higher levels of the AEC2 maturation gene set, again suggesting an inverse relationship between AEC2 maturation and proliferation (Figure 3G). Last, cultured AEC2s expressed a higher number of genes when compared with preculture 1° AEC2s (Figure 3H).

**Figure 4:**
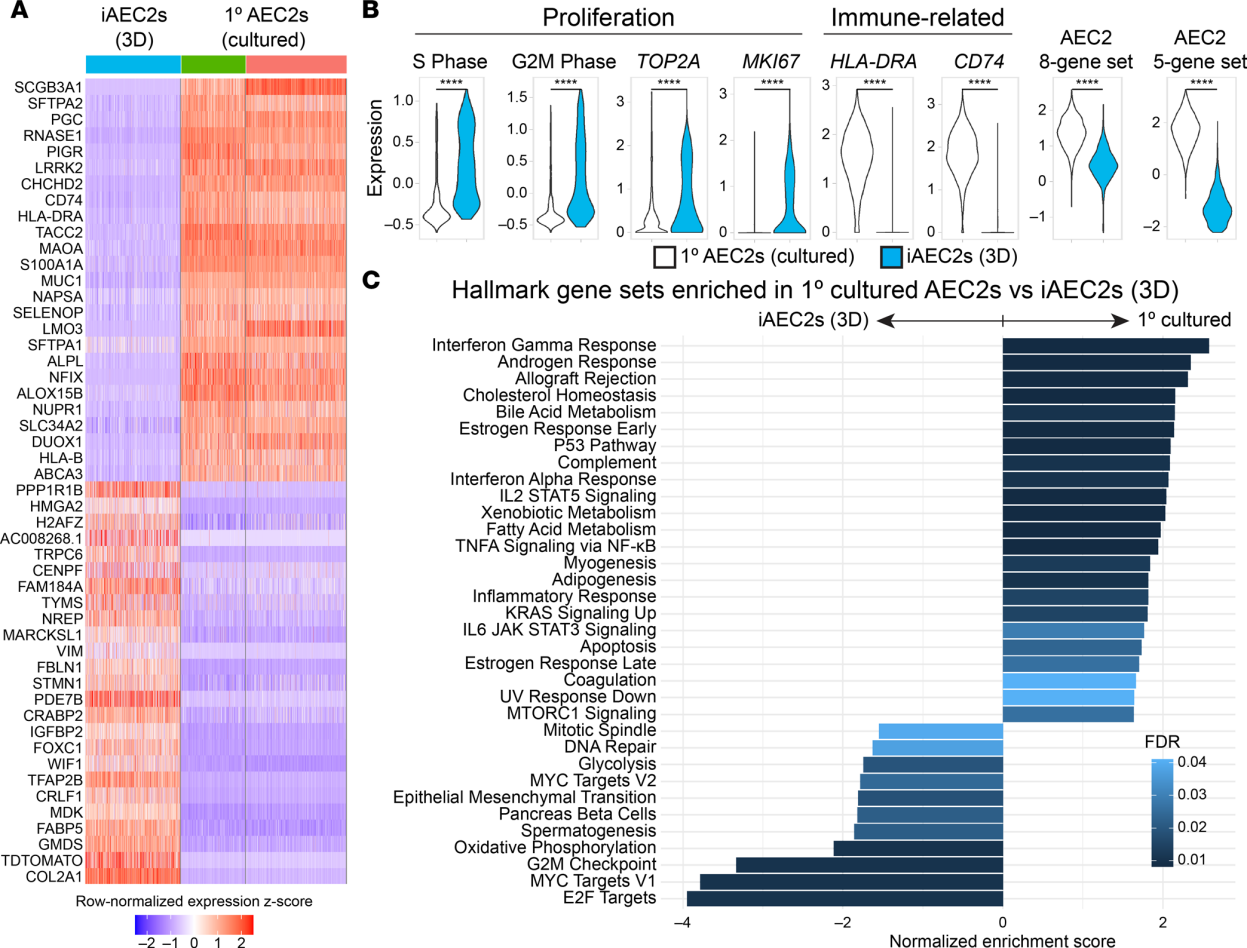
Pairwise single-cell transcriptomic comparisons of 1° cultured AEC2s versus iAEC2s. (**A**) Heatmap of top 25 downregulated and top 25 upregulated genes comparing feeder-free iAEC2s to cultured 1° AEC2s by scRNA-Seq (ranked by average log fold change, FDR < 0.05; row-normalized expression *z* scores). (**B**) Violin plots showing normalized expression for indicated genes, gene sets, or cell cycle phase in 1° cultured AEC2s versus iAEC2s by scRNA-Seq. ****P < 0.0001 by unpaired, 2-tailed Student’s *t* test. (**C**) Gene set enrichment analysis (GSEA, camera using Hallmark gene sets) of differentially regulated gene sets in cultured 1° AEC2s versus feeder-free (3D) iAEC2s (FDR < 0.05).

Comparing feeder-free iAEC2s to cultured 1° AEC2s, we found the distal lung progenitor marker, *HMGA2*, as well as Wnt target gene *WIF1*, to be in the top 25 transcripts differentially upregulated in iAEC2s (Figure 4A). In addition, consistent with their faster growth kinetics and greater ability to be passaged (Figure 1), iAEC2s compared with cultured 1° AEC2s expressed significantly more *MKI67*, *TOP2A*, and gene sets associated with active (S/G2/M phases) cell cycle (Figure 4B). Consistent with this paradigm and the observation that 1° AEC2s are less proliferative than iAEC2s, we found markers of AEC2 differentiation and maturation (*SFTPA2*, *NAPSA*, *SLC34A2*, and *ABCA3*) to be in the top 25 most differentially upregulated transcripts in cultured 1° AEC2s compared with feeder-free iAEC2s. Gene set enrichment analysis (GSEA) indicated p53 signaling among the top upregulated pathways in cultured 1° AEC2s versus iAEC2s and cell cycle pathways (E2F, MYC, G2M checkpoint signaling) among the top downregulated (Figure 4C), consistent with their less active cell cycle and less proliferative state than iAEC2s. Similarly, expression of a p53 module was enriched in cultured 1° AEC2s compared with iAEC2s (Supplemental Figure 3).

**Figure 5:**
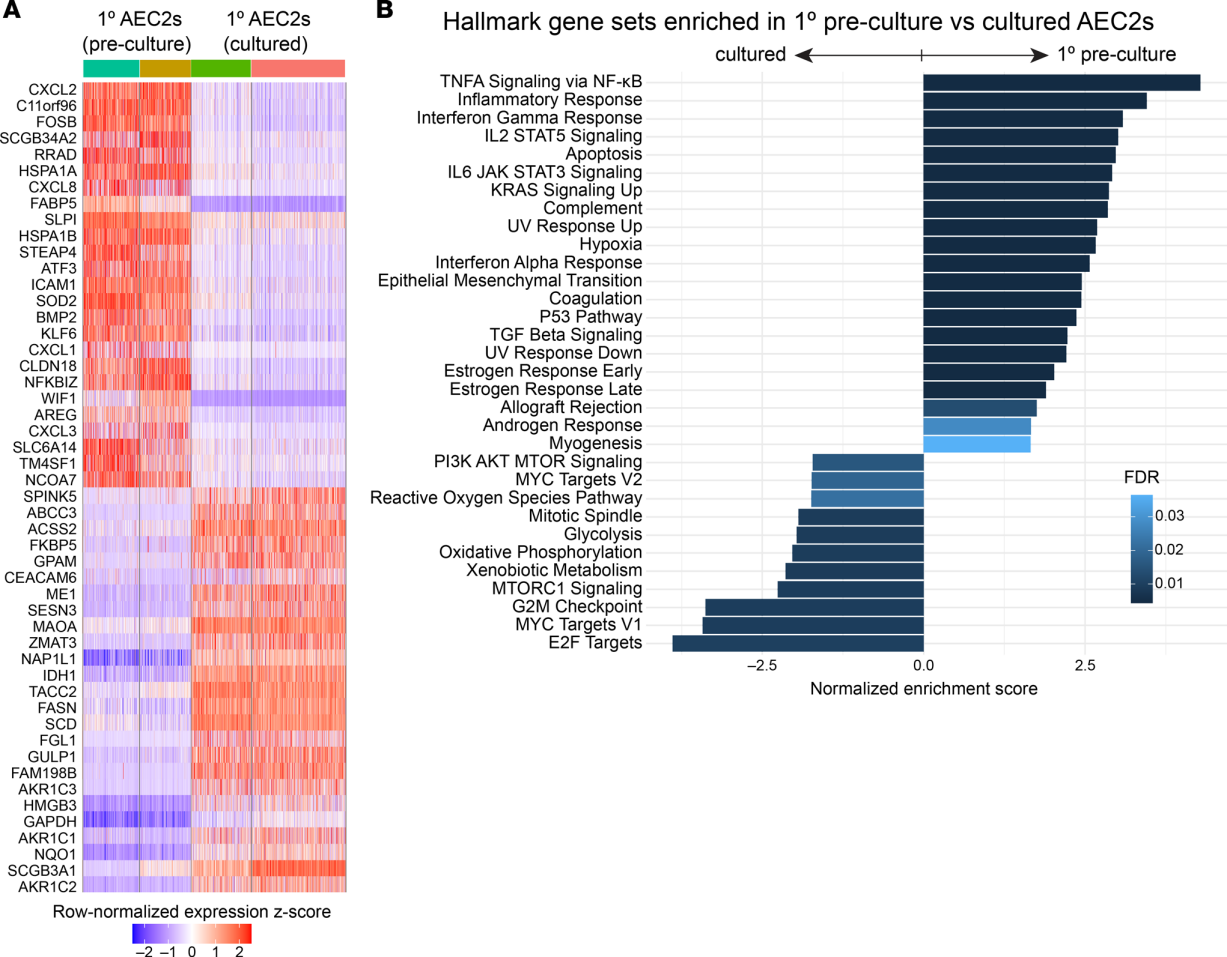
Pairwise single-cell transcriptomic comparisons of preculture 1° AEC2s versus cultured AEC2s. (**A**) Heatmap of top 25 upregulated and top 25 downregulated genes comparing preculture 1° AEC2s to cultured 1° AEC2s by scRNA-Seq (ranked by average log fold change, FDR < 0.05; row-normalized expression *z* scores). (**B**) GSEA (camera using Hallmark gene sets) of differentially regulated gene sets in preculture 1° AEC2s versus cultured AEC2s (cultured 1° AEC2s and feeder-free iAEC2s combined; FDR < 0.05).

Among the several additional pathways enriched in cultured 1° AEC2s versus iAEC2s, there were multiple immune signaling pathways, including NF-κB, IFN-γ, IL-2/STAT5 signaling, and IL-6/JAK/STAT3 signaling (Figure 4C). Most notably, transcripts encoding MHC class I and II members (*HLA-B*, *HLA-DRA*, and *CD74*) were in the top 25 transcripts most downregulated in iAEC2s compared with cultured 1° AEC2s (Figure 4, A and B), consistent with our published (13) finding that iAEC2s express significantly fewer pathways and genes associated with immune maturation compared with 1° AEC2s. Some loss of immune-related transcripts was a consistent feature across culture platforms, including in cultured 1° cells. For example, comparing preculture 1° AEC2s and their cultured progeny, we found a number of immune-related transcripts (*CXCL1*, *CXCL2*, *CXCL3*, *CXCL8*, *NFKBIZ*) to be in the top 25 transcripts differentially downregulated with culture (Figure 5A). Consistent with this, GSEA of preculture 1° versus all cultured AEC2s (1° AEC2s and iAEC2s combined) identified a number of immune-related pathways as being significantly downregulated in cultured cells, whereas proliferation-related pathways were enriched in cultured AEC2s (Figure 5B). The Wnt target genes *WIF1* and *TM4SF1*, a conserved cell surface marker of the previously described Wnt-responsive alveolar epithelial progenitors (12), were also among the top differentially downregulated transcripts after culturing 1° AEC2s (Figure 5A); however, the Wnt target gene *AXIN2* was upregulated (Supplemental Figure 4). Thus, a consistent pattern among Wnt-related genes was not observed across samples, and the differential regulation of some but not all Wnt-responsive genes in cultured versus preculture AEC2s could reflect the presence of the Wnt agonist CHIR99021 in the culture medium.

### Human AEC2s cultured in CK+DCI do not give rise to AEC1s.

**Figure 6:**
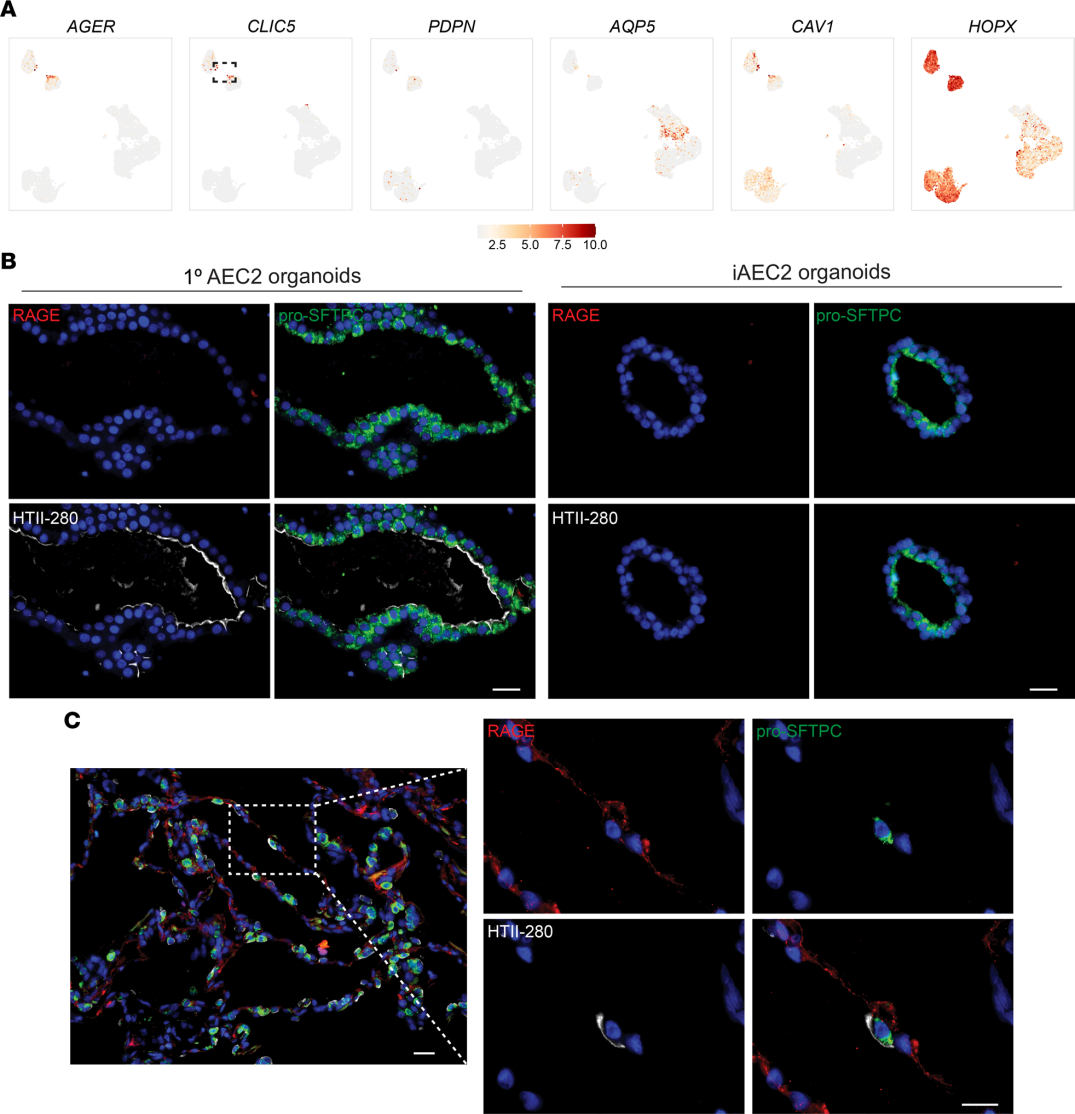
Absence of expression of the AEC1 molecular phenotype in cultured human AEC2s. (**A**) Normalized gene expression overlaid on UMAP plots for the indicated transcripts. Dashed gate shows the few 1° AEC1s. (**B**) Representative immunofluorescence microscopy of 1° AEC2 and iAEC2 organoids cultured in CK+DCI medium stained for RAGE (red), pro-SFTPC (green), HTII-280 (white), and DNA (Hoechst, blue). (**C**) Representative immunofluorescence microscopy of control adult human lung sections stained for the same markers shown in **B**. (**B** and **C**) Scale bars: 25 μm.

Next, we assessed whether there was emergence of AEC1s in our cultured AEC2s or iAEC2s. Notably, there are few, if any, reports of entirely specific AEC1 marker genes whose expression in the human lung has been validated to unambiguously define the cell type. This contrasts with mouse lungs, where broad literature suggests several markers, including *Hopx*, that have been validated as being able to distinguish adult AEC1s from AEC2s (41–43). Thus, we selected multiple gene markers to screen for human AEC1s in our data sets. Using expression levels of a number of genes associated with AEC1s (*AGER*, *CLIC5*, *PDPN*, *AQP5*, *CAV1*, *HOPX*) (42, 44–48) (Figure 6A) or immunostaining for AEC1 markers such as RAGE (Figure 6B), we found no evidence that human 1° AEC2s or iAEC2s differentiated to yield bona fide AEC1s when cultured either alone or in the presence of stromal support cells. The presence of a few AEC1s (coexpressing all canonical AEC1 markers, including *AGER*, *CLIC5*, *CAV1*, *EMP2*, and *PDPN*) in the HTII-280–sorted preculture 1° AEC2 samples (Figure 6A) as well as RAGE-positive AEC1s in immunostained control lung sections (Figure 6C) provided positive controls for expression levels of these markers and further supported our interpretation that there was little to no detectable expression of a comparable AEC1 program in any of our cultured 1° AEC2s or iAEC2s, which is in agreement with previous studies (10, 13). Given that Wnt and KGF (12, 49–58) as well as steroids and cAMP (59, 60) are factors known to promote maintenance of the AEC2 phenotype and proliferation of AEC2s in culture (12, 49–58), we cultured 1° AEC2s in 2 different media (3D medium and SAGM) and iAEC2s in media without CHIR99021 and/or KGF and assessed for the presence of AEC1-like cells by RT-qPCR and immunostaining (Supplemental Figure 5). Robust *AGER* expression (compared to control lung tissue expression levels) was not detected in any condition. While some conditions did have some detectable expression of low levels of *AGER* (compared to control lung), no condition was consistently associated with expression of multiple AEC1 transcripts, such as both *AGER* and *CAV1* (Supplemental Figure 5A). Similarly, immunostaining of 1° AEC2s cultured in 3D medium or SAGM for the AEC1 marker RAGE did not reveal any positive cells (Supplemental Figure 5B). Taken together, these data suggest that none of the AEC2 culture models evaluated in this study yield cell types whose transcriptomes reflect mature human AEC1s.

### Emergence of a transitional cell state.

**Figure 7:**
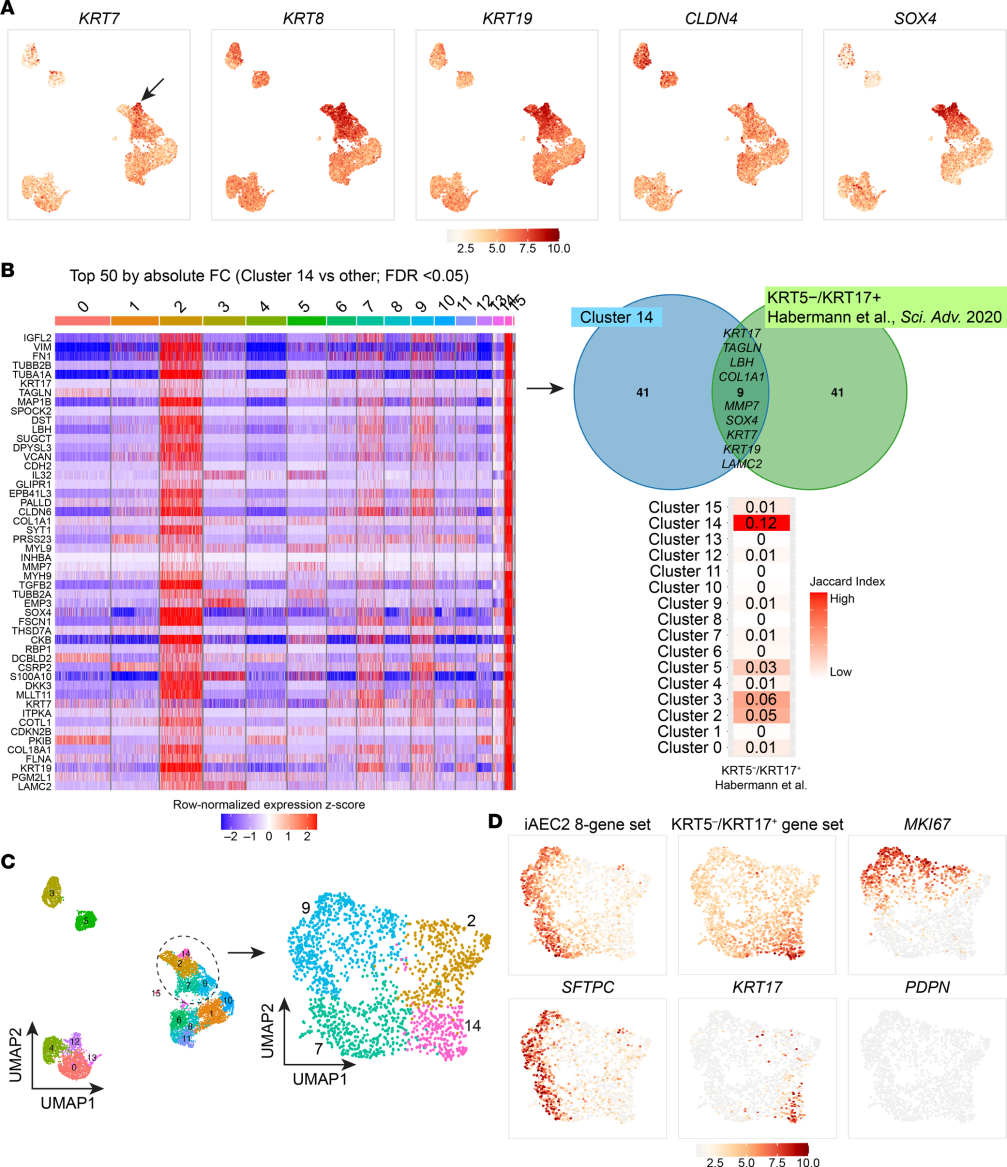
Emergence of a transitional cell state from iAEC2s cocultured with MRC5 fibroblasts. (**A**) Normalized gene expression overlaid on UMAP plots for the indicated transcripts. Arrow indicates *KRT7*^hi^ cells. (**B**) Heatmap of top 50 differentially upregulated genes comparing cluster 14 versus other clusters by scRNA-Seq (ranked by absolute fold change, FDR < 0.05; row-normalized expression *z* scores). Venn diagram shows that 9 of the top 50 differentially upregulated genes in this cluster have previously been associated with a human KRT5^–^KRT17^+^ transitional epithelial cell cluster (30). A row-normalized Jaccard index was calculated between clusters identified in the current study and the KRT5^–^KRT17^+^ transitional epithelial cell cluster (30). (**C**) Louvain clustering of the sample of iAEC2s cocultured with MRC5s maintaining original cluster identity. (**D**) Normalized gene expression overlaid on UMAP plots for the indicated transcripts or gene sets.

**Figure 8:**
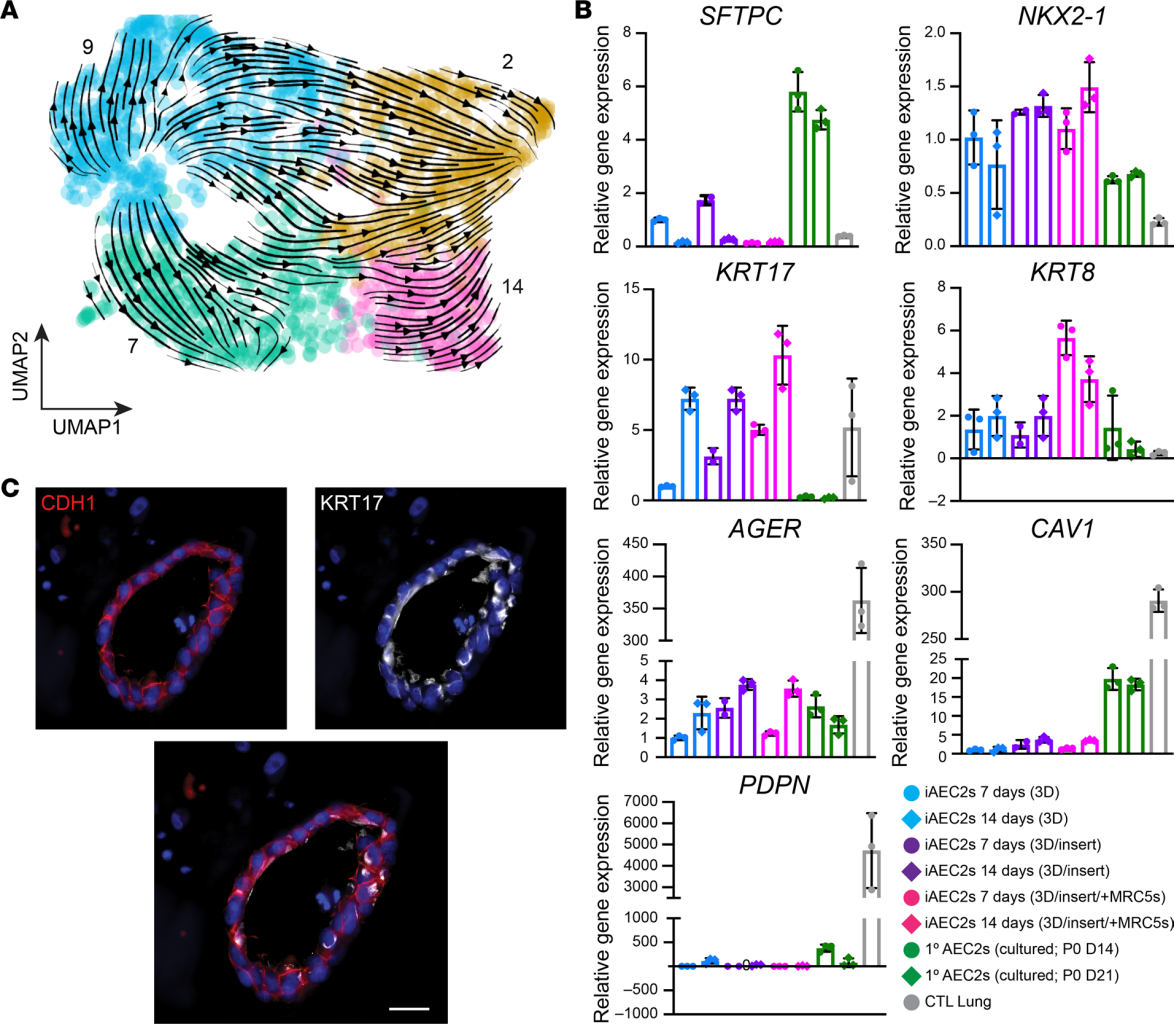
Characterization and kinetics of transitional state marker gene expression. (**A**) RNA velocity analysis indicates that cluster 14 cells (enriched in the KRT5^–^KRT17^+^ gene set; Figure 7D) arise from iAEC2s over time. (**B**) RT-qPCR showing fold change in gene expression in the indicated samples compared with iAEC2s cultured in feeder-free conditions for 7 days. Control samples are an adult human distal lung explant (CTL Lung). (**C**) Representative immunofluorescence microscopy of iAEC2s cocultured with MRC5 fibroblasts and stained for E-cadherin/CDH1 (red), KRT17 (white), and DNA (Hoechst, blue). Scale bars: 25 μm.

Recent studies have described a transitional cell epithelial state detected in injured distal lungs, characterized by expression of a variety of markers not normally expressed in AEC2s and implicated by some authors in the pathogenesis of IPF (24, 25, 31). We thus screened our various samples for the presence of any cells expressing these newly described transitional markers. We found a subset of iAEC2s after coculturing with MRC5 fibroblasts (Figure 2B; cluster 14) was highly enriched for *KRT17* (Figure 2E) and other markers (*KRT7*, *KRT8*, *KRT19*, *CLDN4*, *SOX4*) described in this transitional cell state (Figure 7, A and B). Nine of the top 50 differentially upregulated genes in this cluster have previously been associated with a human *KRT5^–^KRT17^+^* transitional epithelial cell cluster identified in lungs of patients with end-stage IPF (30) (Figure 7B). Using the Jaccard index (61, 62), we performed comparisons between the clusters identified in the current study and the *KRT5^–^KRT17^+^* transitional cell cluster described in Habermann et al. (30) and found that cluster 14 (and related cluster 2) had the most similarity to the *KRT5^–^KRT17^+^* transitional cell state (Figure 7B). Focusing on the sample containing these clusters (iAEC2s cocultured with MRC5s; Figure 7C), iAEC2-derived cells expressing the *KRT5^–^KRT17^+^* gene set demonstrated lower expression of both *SFTPC* and members of an 8-gene signature that defines AEC2s (Figure 7D) (36). These cells did not express *MKI67*, suggesting they were quiescent (Figure 7D). In contrast, cluster 2 represented a more proliferative subset of the transitional cell state based on higher *MKI67* expression (Figure 7D). Cluster 2 cells were enriched in expression of *NOTUM*, *NKD1*, *CST1*, *MFAP2*, *LEF1*, and *CD8B* (Supplemental Figure 2) and clustered closely together with cluster 14 cells by UMAP analysis (Figure 7C). To better map the origin of *KRT5^–^KRT17^+^* epithelial cells and since these cells presumably emerged from the starting iAEC2 population at some point over 7 days of coculture with MRC5s, we performed RNA velocity analysis, which was consistent with the interpretation that iAEC2s gave rise to the *KRT5^–^KRT17^+^* clusters of cells over time (clusters 2 and 14; Figure 8A). To assess whether these cells might fully transition to AEC1s at a later time, we repeated cultures of iAEC2s alone versus iAEC2s cultured with supporting cells and evaluated their gene expression weekly for 2 weeks, compared with cultured 1° AEC2 controls. We found that expression of both *KRT17* and *KRT8* increased over time in cultures of iAEC2s, particularly those cultured with MRC5 cells (Figure 8B). Cultured iAEC2s in these conditions again did not exhibit evidence of AEC1 differentiation based on low or no expression of *AGER*, *PDPN*, or *CAV1* over the 2-week culture period (Figure 8B). Immunofluorescence staining demonstrated the presence of KRT17 expression at the protein level in a subset of epithelial cells after coculturing of iAEC2s with MRC5 fibroblasts (Figure 8C). These data support the emergence of a transitional/intermediate epithelial cell state in iAEC2/fibroblast cocultures that resembles transitional *KRT5^–^KRT17^+^* cells described in IPF lung tissue by Habermann et al. (30).

## Discussion

Our results herein suggest that cultured 1° AEC2s and iAEC2s maintain an AEC2-like phenotype in vitro and their transcriptomes at single-cell resolution exhibit similarities to and important differences from 1° preculture AEC2s. Most notably, we find a continuum of progressively more proliferative states (from preculture 1° to cultured 1° to iAEC2s) correlating with greater ability to be passaged and inversely associated with AEC2 maturation state (Figures 3 and 4). Furthermore, our results reveal that immune-related programs, such as those involving MHC genes, are expressed at progressively lower levels in cultured AEC2s (decreasing from preculture 1° AEC2s to cultured 1° AEC2s to iAEC2s), suggesting that in vivo AEC2s are immunologically more mature than cultured cells.

Importantly, our results suggest that despite their limitations cultured 1° AEC2s and iAEC2s maintain key components of the human AEC2 program, and thus it is likely that both cell populations can serve as in vitro models of the human lung epithelium for disease modeling with relevant benefits or limitations that should be considered when selecting each type of model system. The different characteristics of 1° AEC2s (higher expression of immune-related transcripts) and iAEC2s (patient specificity, greater proliferation and ability to be passaged) indicate that each model can be applied in different settings. For example, cultured 1° AEC2s with their higher expression of immune-related genes and pathways may serve as preclinical disease models to study the responses of the alveolar epithelium to infectious agents, such as SARS-CoV-2, in situations where immune maturation may be particularly relevant (63). In cases where high levels of expression of MHC genes are not required, other human AEC2-specific responses to infectious agents, including SARS-CoV-2, may be studied in either cultured cell type, since both 1° cultured AEC2s and iAEC2s express a wide variety of receptors required for viral entry, such as ACE2 and TMPRSS2 (63, 64). Cultured 1° AEC2s may also be a better model for studying the epithelial-mesenchymal interactions at play in fibrotic lung diseases, since iAEC2s cocultured with MRC5 fibroblasts lost the AEC2 phenotype. However, the emergence of a transitional cell phenotype in these culture conditions could prove beneficial for IPF disease modeling. On the other hand, patient-specific iAEC2s can be used as a human preclinical disease model when unable to access 1° AEC2s at early disease stages or when the application of invasive procedures required to isolate adequate numbers of 1° AEC2s is prohibitive, such as in the case of severe interstitial lung disease (37). Patients with advanced lung disease are likely to have AEC2s with transcriptomic and epigenetic programs that are secondarily perturbed by drugs, infections, ventilators, or other tertiary insults that may be difficult to distinguish from earlier disease-initiating mechanisms. Since iAEC2s are derived from iPSCs generated by reprogramming, which erases the starting epigenome or disease state, patient-specific iAEC2s in most cases will have been “reset” and thus in theory the emergence of disease, potentially including relevant genetic and epigenetic AEC2 programs, may be replayed repeatedly, potentially from disease inception through late stages and drug responses (37). This sequence may be harder to study in cultured 1° AEC2s procured from some patients with advanced disease, particularly if the disease results in a paucity of AEC2s in vivo, or if residual epigenetic perturbations or lasting secondary effects are carried through in any cultured 1° AEC2s from those patients.

In terms of disease-relevant cells that may arise from parental AEC2s, the emergence of so-called transitional cells from the coculture of iAEC2s with mesenchymal cells was an unexpected result with clinically relevant implications. These iAEC2-derived transitional cells appeared to express a transcriptomic phenotype overlapping with the recently described transitional cell states observed in mice (24, 25, 31, 33) and humans (25, 31, 34) during alveolar repair after lung injury and in distal lung tissues of patients with fibrosing illnesses (24, 25, 28–31), suggesting that these culture conditions could be used to better understand these cells’ cellular origin and potential pathogenic or reparative roles.

Cultured 1° AEC2s, compared with iAEC2s, expressed significantly higher levels of transcripts known to be expressed late or postnatally in AEC2 development, also known as AEC2 maturation markers (Figure 4). These markers include surfactant-encoding transcripts known to be expressed late in AEC2 development, such *SFTPA1* and *SFTPA2* (36). In contrast, early surfactants, *SFTPC* and *SFTPB*, were expressed at high levels in both cultured 1° AEC2s and iAEC2s. iAEC2s have already been successfully employed to study the processing of normal and mutant SFTPB and SFTPC proteins, including disease modeling using patient-specific mutant iAEC2s and their gene-edited progeny (13, 37); however, for studies focused on understanding the biology of late surfactants, such as SFTPA1 and SFTPA2, investigators may choose 1° AEC2s rather than iAEC2s, at least when using the culture conditions employed in this report.

Whether the expression of maturation markers can be further augmented in iAEC2s, to the levels we found expressed in 1° cells, is an area of ongoing research. For example, we recently reported that transitioning iAEC2s to 2D air-liquid interface culture conditions results in decreased proliferation and increased maturation, exemplified by augmented expression of both early and late surfactants (*SFTPC*, *SFTPA1*, *SFTPA2*, and secretion of tubular myelin) (64, 65). It remains to be seen, however, whether this level of augmented maturation is able to reach the high levels of expression exhibited by 1° AEC2s in the scRNA-Seq profiles we present here.

A particular hurdle that has limited research using human AEC2s in the past has been the inability to stably expand 1° AEC2s in culture, which would be required for gene editing studies, high-throughput drug screens, or future cell-based therapies. Our results indicate that iAEC2s provide a potential solution since they can be indefinitely expanded in 3D cell culture and exhibit a more proliferative state than 1° AEC2s either before or after culture. Indeed, we have previously shown that more than 10^23^ iAEC2s can be derived per input patient-specific iAEC2 via serial passaging over a 300-day culture period (37). Although 1° AEC2s could not be serially passaged extensively in our current culture conditions, since the preparation of this manuscript 3 reports have published methods for maintaining 1° human AEC2s in extensively self-renewing, feeder-free cultures (14–16). Future work will be required to compare fresh/uncultured 1° AEC2s to their progeny expanded in these new conditions as well as to iAEC2s to understand whether the transcriptomic programs and functional repertoire of AEC2s differ from the cells profiled in our conditions.

Many human iPSC-based model systems, such as those designed to derive cardiomyocytes or hepatocytes, are characterized by the presence of embryologically immature cells relative to their 1° control cell counterparts (66, 67). It remains unclear, however, whether the lower levels of maturation markers expressed in iAEC2s compared with 1° cells in our studies should be interpreted as a lack of developmental maturation versus the acquisition in culture of a highly proliferative state, such as one that might occur in postnatal distal lung tissue in vivo in response to injury. Indeed, the use of enzymes to digest tissues or epithelial spheres into single-cell suspensions for culture likely represents a significant injury to which AEC2s must respond if they are to survive and proliferate in our culture conditions. As in prior reports (39, 40), our profiles indicate an inverse relationship between AEC2 proliferation and maturation states. However, the lack of current in vivo benchmarking scRNA-Seq data sets from human fetal developing and postinjury adult lungs limits a definitive determination of which in vivo stages or states (fetal/developmental or adult postinjury, for example) are most similar to either iAEC2s or 1° cultured AEC2s. Intense research efforts, such as LungMAP, that are designed to prepare these types of in vivo benchmarks should help address this ongoing question.

Finally, a controversy raised by our findings is whether human AEC2s generate bona fide AEC1s in cell culture, as has been extensively demonstrated with mouse AEC2s (10, 22, 24, 25). Despite recent studies (12, 14, 15, 63) suggesting human AEC2 to AEC1 differentiation in vitro based on expression of a few selected markers, such as HTI-56 and additional bulk transcriptomic profiles of in vitro human alveolar cell derivatives (44, 68, 69), it remains uncertain how closely these cultured AEC1-like cells resemble their in vivo counterparts, if compared head-to-head. Gotoh and colleagues recently showed generation of AEC1-like cells from iAEC2s in feeder-dependent cultures (70). However, comprehensive profiling of our cultures at single-cell resolution, based on expression of a set of markers and confirmed by immunostaining, reveals little evidence of cultured adult human AEC2s giving rise to bona fide AEC1s, at least under our culture conditions, in agreement with some prior reports studying 1° or iPSC-derived human cells (10, 13). Given that AEC1s have been recently identified as an active signaling hub in the alveolus (71), the absence of AEC1-like cells from these cultures may have implications when these models are used for the study of lung development and repair following injury. An additional limitation of our study is the relatively small sample size of 1° AEC2 donors (*n* = 5).

In summary, our results suggest that culturing human AEC2s has defined and quantifiable impacts on their transcriptomic programs and proliferative states. By profiling these cells head-to-head and at single-cell resolution, we thus establish an interactive online tool (https://crem-bu.shinyapps.io/scRNAseq-AEC2-comparisons/) to interrogate gene expression in each population, and we provide comparisons of preculture 1° AEC2s, cultured 1° AEC2s, and iAEC2s, revealing similarities and differences and suggesting that both cultured 1° AEC2s and iAEC2s can serve as in vitro models to study the role of human AEC2s in the pathobiology of the distal lung.

## Methods

### iPSC line generation and maintenance.

The SPC2 iPSC line carrying a SFTPC^tdTomato^ reporter (SPC2-ST-B2 clone), as previously detailed (37), was used in this study. iPSCs used in this study demonstrated a normal karyotype when analyzed by G-banding and/or array Comparative Genomic Hybridization (Cell Line Genetics). iPSCs were maintained in feeder-free conditions, on growth factor–reduced Matrigel (Corning) in 6-well tissue culture dishes (Corning), and in mTeSR1 media (StemCell Technologies) using gentle cell dissociation reagent for passaging. Further details of iPSC derivation, characterization, and culture are available for free download at https://crem.bu.edu/cores-protocols/#protocols

### iPSC-directed differentiation into alveolar epithelial type 2 cells (iAEC2s).

To generate iAEC2s, we performed PSC-directed differentiation via definitive endoderm into NKX2-1 lung progenitors using methods we have previously described (13, 37, 38, 72). On day 15 of differentiation, live cells were sorted on a high-speed cell sorter (MoFlo Astrios EQ) to isolate NKX2-1^+^ lung progenitors based on CD47^hi^CD26^–^ gating (73). Sorted lung progenitors were resuspended in undiluted growth factor–reduced 3D Matrigel (Corning) at a density of 400 cells/μL, and distal/alveolar differentiation of cells was performed in CK+DCI medium, consisting of complete serum-free differentiation medium base supplemented with 3 μM CHIR99021, 10 ng/mL recombinant human KGF (CK), and 50 nM dexamethasone (Sigma), 0.1 mM 8-Bromoadenosine 3′,5′-cyclic monophosphate sodium salt (Sigma), and 0.1 mM IBMX (Sigma) (DCI). The resulting epithelial spheres were passaged without further sorting on day 28 of differentiation followed by a brief period of CHIR99021 withdrawal (days 31–35, Figure 1E) to achieve iAEC2 maturation, as previously shown (13). To establish pure cultures of iAEC2s, cells were purified by FACS to isolate SFTPC^tdTomato+^ cells on days 41 and 69 of differentiation. iAEC2s were then maintained through serial passaging as self-renewing monolayered epithelial spheres (“alveolospheres”) by plating in 3D Matrigel (Corning) droplets at a density of 400 cells/μL with refeeding every other day with CK+DCI medium, according to our published protocol (38). iAEC2 culture quality and purity were monitored at each passage by flow cytometry, with more than 95% of cells expressing SFTPC^tdTomato^ over time, as we have previously detailed (36, 37).

For the culture of iAEC2s on cell culture inserts, iAEC2s were resuspended in CK+DCI medium at a density of 10 × 10^3^ cells/50 μL and then mixed with 3D Matrigel (Corning) or with 3D Matrigel (Corning) containing MRC5 fibroblasts at a density of 50 × 10^3^ cells/50 μL. Then, 100 μL of either condition was seeded on a 0.4 mm–pore cell culture insert in a 24-well supported format (Corning). Next, 500 μL CK+DCI medium was added to the bottom chamber. Medium was changed every other day. Cultures were maintained at 37°C in a humidified incubator (5% CO_2_).

### Lung tissue processing.

Sub–transplant-quality human lung specimens, with no history of respiratory disease, were obtained through the International Institute for the Advancement of Medicine in compliance with consent procedures developed by the International Institute for the Advancement of Medicine and approved by the Boston Children’s Hospital and Cedars-Sinai Medical Center Institutional Review Boards. Upon arrival the tissue was washed with DMEM/F12 (Corning) supplemented with antibiotic/antimycotic solution (Sigma). Approximately 1–2 cm^3^ pieces of distal lung were finely minced and mixed with 1 mL freezing medium consisting of FBS (Thermo Fisher Scientific) with 10% DMSO (Sigma) and frozen in liquid nitrogen until needed.

### Flow cytometry and FACS.

Immunostaining of cells in single-cell suspension with antibodies against CD47 (BioLegend) and CD26 (BioLegend) and FACS gating of the CD47^hi^CD26^–^ population for purification of lung progenitors were performed as previously described (38, 73). Gating was based on isotype-stained controls or in the case of SFTPC^tdTomato^ on nonlung endoderm outgrowths (day 15–sorted CD47^lo^ cells). Preparation of single-cell suspensions of 3D Matrigel–embedded iAEC2s for flow cytometry and FACS was achieved by incubation with 2 mg/mL dispase (Thermo Fisher Scientific) for 30–60 minutes at 37°C and subsequent incubation with 0.05% trypsin for 12–15 minutes at 37°C, as previously described (38). Cells were washed with media containing 10% FBS (Thermo Fisher Scientific). Harvested cells were centrifuged at 300*g* for 5 minutes at 4°C and resuspended in FACS buffer consisting of Hanks balanced salt solution (HBSS, Thermo Fisher Scientific) supplemented with 2% FBS and 10 μM Y-27632 (Tocris) and stained with calcein blue AM (Thermo Fisher Scientific) for dead cell exclusion. Live cells were sorted on a high-speed cell sorter (MoFlo Astrios EQ) at the Boston University Medical Center Flow Cytometry Core Facility.

The 3D Matrigel in the 1° AEC2 and iAEC2 cultures on inserts was dissolved by adding 150 μL of dispase (Corning) to the apical chamber of the inserts and incubating for 1 hour at 37°C followed by 0.25% trypsin/EDTA (Invitrogen) for 5 minutes at 37°C. The reaction was quenched with 10% FBS DMEM/F12. Pellets were resuspended in staining buffer consisting of PBS supplemented with 2% FBS and 2 mM EDTA and stained using antibodies against EPCAM (9C4) and DAPI for viability. On the day of encapsulation (day 0, Figure 1), 1 vial of frozen tissue per sample was thawed at 37°C and incubated in HBSS (Thermo Fisher Scientific) containing Liberase (50 mg/mL, Sigma) and DNase 1 (25 mg/mL, Thermo Fisher Scientific) at 37°C for 50 minutes with gentle shaking. Dissociated cells were passed through a series of cell strainers of decreasing pore sizes from 500 μm to 70 μm. Pellets were resuspended in staining buffer and stained using antibodies against EPCAM (9C4), HTII-280 (Terrace Biotech), and DAPI (Sigma) for viability determination. Live cells were sorted for EPCAM^+^ using a FACSAria Fusion (BD); HTII-280 status was used for analysis.

Flow cytometry staining was quantified using the Stratedigm S1000EXI and analyzed with FlowJo v10.6.2 (Tree Star Inc). Flow cytometry plots shown represent single cells after forward-scatter/side-scatter gating to remove debris as we have previously detailed (38). Specifics of antibodies used are detailed in Supplemental Table 1.

### Primary (1°) AEC2 cell isolation.

One vial of frozen tissue per sample was thawed at 37°C and incubated in HBSS (Thermo Fisher Scientific) containing Liberase (50 mg/mL) and DNase 1 (25 mg/mL) at 37°C for 50 minutes with gentle shaking. Dissociated cells were passed through a series of cell strainers of decreasing pore sizes from 500 μm to 70 μm. Cell pellets were resuspended in staining buffer, and single-cell suspension preparations were labeled using antibodies against EPCAM (9C4), CD31 (WM59), CD45 (HI30), rat anti–mouse IgM (BioLegend), HTII-280 (Terrace Biotech), and DAPI (Sigma) for viability determination. Labeled cells were sorted using a FACSAria Fusion (BD).

### Generation of 1° AEC2 organoids.

CD31^–^CD45^–^EPCAM^+^HTII-280^+^ alveolar epithelial cells were resuspended in CK+DCI medium, 3D medium (DMEM/F12 supplemented with 10% FBS, penicillin/streptomycin 1,000 U/mL, 1 mM HEPES, 1 mM l-glutamate, and insulin/transferrin/selenium from Sigma), or SAGM as shown at a density of 5 × 10^3^ cells/50 μL and then mixed with 3D Matrigel (Corning) containing MRC5 fibroblasts (ATCC CCL-171) at a density of 50 × 10^3^ cells/50 μL. A total of 100 μL of the suspension was seeded on a 0.4 mm–pore cell culture insert in a 24-well supported format (Corning). After polymerization of Matrigel, 500 μL of CK+DCI medium was added to the bottom chamber. Medium was supplemented with 10 μM Y-27632 for the first 48 hours. Medium was changed every other day. Cultures were maintained at 37°C in a humidified incubator (5% CO_2_).

### Passaging of 1° AEC2 organoids.

For passaging the alveolar organoids, 3D Matrigel was dissolved by adding 150 μL of dispase (Corning) to the apical chamber of the inserts and incubating for 1 hour at 37°C followed by 0.25% trypsin/EDTA (Invitrogen) for 5 minutes at 37°C. The reaction was quenched with 10% FBS DMEM/F12. Pellets were resuspended in staining buffer, and single-cell suspensions were stained using antibodies against EPCAM (9C4), HTII-280 (Terrace Biotech), and DAPI (Sigma) for viability determination. Labeled cells were sorted for EPCAM^+^ using a FACSAria Fusion; HTII-280 expression status was used for analysis. A total of 5 × 10^3^ EPCAM^+^ viable cells were mixed with 5 × 10^4^ MRC5 fibroblasts (ATCC CCL-171) and resuspended in a 50:50 (v/v) ratio of ice-cold 3D Matrigel (Corning) and CK+DCI medium. Next, 100 μL of the suspension was seeded on a 0.4 mm–pore cell culture insert in a 24-well supported format (Corning). After polymerization of Matrigel, 500 μL of CK+DCI medium was added to the bottom well. Medium was supplemented with 10 μM Y-27632 for the first 48 hours. Medium was changed every other day.

### Immunofluorescence microscopy.

Images of cultured 3D Matrigel–embedded iPSC-derived epithelial spheres were taken on a Keyence BZ-X700 fluorescence microscope. *Z*-stack images were processed using full focus image analysis using Keyence software.

For formalin fixation and paraffin embedding, cultures were fixed by adding 200 μL of 10% formalin to the insert and 500 μL to the bottom chamber and incubating overnight at 4°C. Fixed samples were cut out of cell culture inserts and embedded in HistoGel (Thermo Fisher Scientific), then processed for paraffin and sectioning. Sectioned lung tissues or organoids were immunostained following antigen retrieval with citric acid buffer (Thermo Fisher Scientific). Blocking was performed with 5% normal donkey serum in 0.2% Triton-X/PBS at room temperature for 60 minutes. Primary antibodies were incubated overnight at 4°C at the indicated dilutions: pro-SFTPC (1:1,000, Seven Hills Bioreagents), HTII-280 (1:100, Terrace Biotech), KRT8 (1:100, Abcam), KRT17 (1:100, Abcam), RAGE (1:100, R&D Systems), and E-Cadherin/CDH1 (1:100, Thermo Fisher Scientific). Alexa Fluor–conjugated secondary antibodies (1:500, Thermo Fisher Scientific) were incubated at room temperature for 2 hours. After antibody staining, nuclei were stained with DAPI (Sigma) and mounted using Molecular Probes ProLong Gold Antifade Mountant (Thermo Fisher Scientific). Specifics of antibodies used are detailed in Supplemental Table 2. Fluorescence images were acquired using a fluorescence microscope (Nikon Eclipse 90i). All the images were further processed with Fiji software.

### Western blot analyses.

Cell pellets were treated with lysis buffer (RIPA buffer and 1× Roche Complete Protease Inhibitor cocktail) and incubated on ice for 30 minutes. Cellular debris was cleared by centrifugation at 15,000*g* for 20 minutes at 4°C and supernatants were harvested. Protein concentration was measured using Bio-Rad DC Protein Assay. Cell lysates were resolved on precast 12% Bis-Tris NuPAGE gels (Invitrogen), transferred to PVDF membranes (Bio-Rad), and blotted with primary antibodies (see below and Supplemental Table 2) followed by species-specific HRP-conjugated secondary antibody. Primary antibodies (see Supplemental Table 2) include surfactant protein B (PT3, a rabbit polyclonal antibody against bovine SFTPB (1:2,500) (74), mature SFTPC (1:1,000, Seven Hills Bioreagents), and hexokinase 1 (1:2,500, Proteintech) used as the loading control (75). Visualization was accomplished using the Odyssey Imaging System (LICOR Biosciences).

### RT-qPCR.

RNA was extracted by first lysing cells in QIAzol (QIAGEN) and subsequently using the RNeasy Mini Kit (QIAGEN) according to the manufacturer’s protocol. cDNA was generated by reverse transcription of 1 μg RNA from each sample using MultiScribe Reverse Transcriptase (Applied Biosystems). RT-qPCR was performed using TaqMan Fast Universal PCR Master Mix (Thermo Fisher Scientific) and TaqMan (Applied Biosystems) reagents. The cDNA was diluted 1:4 and 2 μL of cDNA was added to each 20 μL (for Applied Biosystems StepOne 96-well system) or 12 μL (for Applied Biosystems QuantStudio7 384-well system) RT-qPCR reaction. Each sample was run in technical duplicates or triplicates for 40 cycles of PCR, and cycle threshold (Ct) values were averaged between replicates for analysis. Relative gene expression, normalized to 18S control, was calculated as fold change in 18S-normalized gene expression, compared to baseline, using the 2^-ΔΔCt^ method. Baseline expression, defined as fold change = 1, was set to 3D Matrigel–cultured iAEC2 levels, or if undetected, a cycle number of 40 was assigned to allow fold change calculations. Adult human lung control RNA was extracted from a healthy donor’s distal lung explant. Primers were all TaqMan probes purchased from Applied Biosystems. Specifics of primers used are detailed in Supplemental Table 3.

### scRNA-Seq and bioinformatic analyses.

scRNA-Seq comparing i) 3D feeder-free iAEC2 cultures; ii) 3D feeder-free iAEC2 cultures on cell culture inserts; iii) 3D iAEC2 insert cultures with MRC5 fibroblasts; iv) preculture 1° AEC2s, defined as freshly isolated, cryopreserved HTII-280^+^ 1° AEC2s FACS-purified; and 5) cultured 1° AEC2s, defined as the P0 1° AEC2 progeny of the preculture 1° AEC2s after 21 days of culturing in CK+DCI medium with MRC5 fibroblasts on cell culture inserts. The iAEC2 sample cocultured with MRC5s on inserts and the 2 cultured 1° AEC2 samples were sorted for viable EPCAM^+^ cells prior to encapsulation. All other samples were sorted for live cells only. To harvest cells of similar confluence despite different outgrowth kinetics of 1° AEC2s versus iAEC2s, iAEC2s were harvested on day 7 after passage, whereas P0 1° AEC2s were harvested on day 21. Single cells were captured for sequencing library preparation using a Chromium (10x Genomics) instrument. scRNA-Seq libraries were prepared according to the Single-Cell 3’ v3 Reagent Kits User Guide (10x Genomics). Cellular suspensions were loaded on a Chromium Controller instrument (10x Genomics) to generate single-cell gel bead-in-emulsions (GEMs). Reverse transcription (GEM-RT) was performed in a Veriti 96-well thermal cycler (Thermo Fisher Scientific). After RT, GEMs were harvested and the cDNAs were amplified and cleaned with SPRIselect Reagent Kit (Beckman Coulter). Indexed sequencing libraries were constructed using the Chromium Single-Cell 3’ Library Kit (10x Genomics) for enzymatic fragmentation, end repair, A-tailing, adapter ligation, ligation cleanup, sample index PCR, and PCR cleanup. The barcoded sequencing libraries were quantified by quantitative PCR using the KAPA Biosystems Library Quantification Kit. Sequencing libraries were loaded on a NextSeq500 (Illumina) with a custom sequencing setting (26 bp for read 1 and 98 bp for read 2), to obtain a sequencing depth of about 50,000 reads/cell. FASTQ files were generated using bcl2fastq v.2.2 and Cell Ranger v.3.0.2. The sequence files were mapped to the human genome reference (GRCh37) including the tdTomato reporter. We used Seurat v.3 to further process the data. We estimated the doublet rate according to the Chromium guidelines, in proportion to the density of cells loaded. These rates were used to flag potential doublets based on their gene and unique molecular identifier counts. Cells with fewer than 800 genes detected were also filtered out, as well as cells with high percentage of counts mapping to mitochondrial genes (thresholds set by manual inspection for each data set, between 25% and 35%). Samples were merged and then normalized using SCTransform, with cell degradation effect regressed out. After an initial linear dimensionality reduction (principal component analysis), we used UMAP to represent the data, and the Louvain algorithm was used for clustering, at a resolution of 1. Differential expression tests between the samples specified in each figure or table were done with Model-based Analysis of Single-cell Transcriptomics (https://github.com/RGLab/MAST; commit ID 339c6a7), applying independent filtering before gene testing (minimum gene detection in 10% of the cells in at least 1 of the populations, minimum average log fold change in expression of 0.25 between the 2 populations). An FDR-adjusted *P* value cutoff of 0.05 was used to determine statistically significant differential gene expression. Gene signature enrichment scores for AEC2 markers and cell cycle stage were computed using Seurat. The significance of the differences in these scores between samples was tested using Welch’s 2-sample *t* tests. For comparing scRNA-Seq data generated in this study with existing data sets, we used matchSCore2 R package.

The scRNA-Seq data discussed in this publication have been deposited in NCBI’s Gene Expression Omnibus (accession number GSE193716). In addition, an online Shiny app tool (https://crem-bu.shinyapps.io/scRNAseq-AEC2-comparisons/) has been established to allow interactive, user-friendly visualizations of gene expression in each population, and worksheets containing the results of differential gene expression between samples are included in Supplemental Table 4.

### Statistics.

Statistical methods relevant to each figure are outlined in each figure legend. In brief, unpaired, 2-tailed Student’s *t* tests were used to compare quantitative analyses comprising 2 groups of *n* = 3 or more samples, or 1-way ANOVAs with multiple comparisons were used to compare 3 or more groups. Further specifics about the replicates used in each experiment are available in the figure legends. In these cases, a Gaussian distribution and equal variance between samples were assumed as the experiments represent random samples of the measured variable. The *P* value threshold to determine significance was set at *P* = 0.05. Data for quantitative experiments are typically represented as the mean with error bars representing the SD or SEM, as specified in the figure legends.

### Study approval.

The SPC2 iPSC line carrying a SFTPC^tdTomato^ reporter (SPC2-ST-B2 clone), as previously detailed (37), was used in this study. The Human Research Protection Office of Washington University School of Medicine, St. Louis, Missouri, USA, approved procurement of dermal fibroblasts for reprogramming with written informed consent. All experiments involving the differentiation of human iPSCs were performed with the approval of the Institutional Review Board of Boston University (protocol H33122). Further details of iPSC derivation, characterization, and culture are available for free download at https://crem.bu.edu/cores-protocols/#protocols

## Author contributions

KDA, CGDA, PP, BRS, CFK, and DNK conceived the work. KDA, CGDA, PP, MFB, BRS, CFK, and DNK designed experiments. KDA, CGDA, CY, PP, JH, KM, CLB, OTH, AM, and BK conducted experiments and analyzed data. CVM, PB, and CY performed bioinformatics analysis. KDA, CGDA, PP, BRS, CFK, and DNK prepared and edited the manuscript. Two co–first authors were assigned reflecting equal contribution to this collaboration. The authorship order was assigned based on consensus of the co–first and co–senior authors. Whereas other authors also made important contributions, the 2 co–first authors made the most significant contributions to complete the manuscript. All authors reviewed and approved the final version prior to submission.

## Supplementary Material

Supplemental data

Supplemental table 4

## Version 1 12/01/2022

In-Press Preview

## Version 2 01/10/2023

Electronic publication
