# Supplementary material for: Culture impact on the transcriptomic programs of primary and iPSC-derived human alveolar type 2 cells
Source: JCI Insight. 2023 Jan 10;8(1):e158937. doi: 10.1172/jci.insight.158937 (PMC9870086; doi:10.1172/jci.insight.158937)

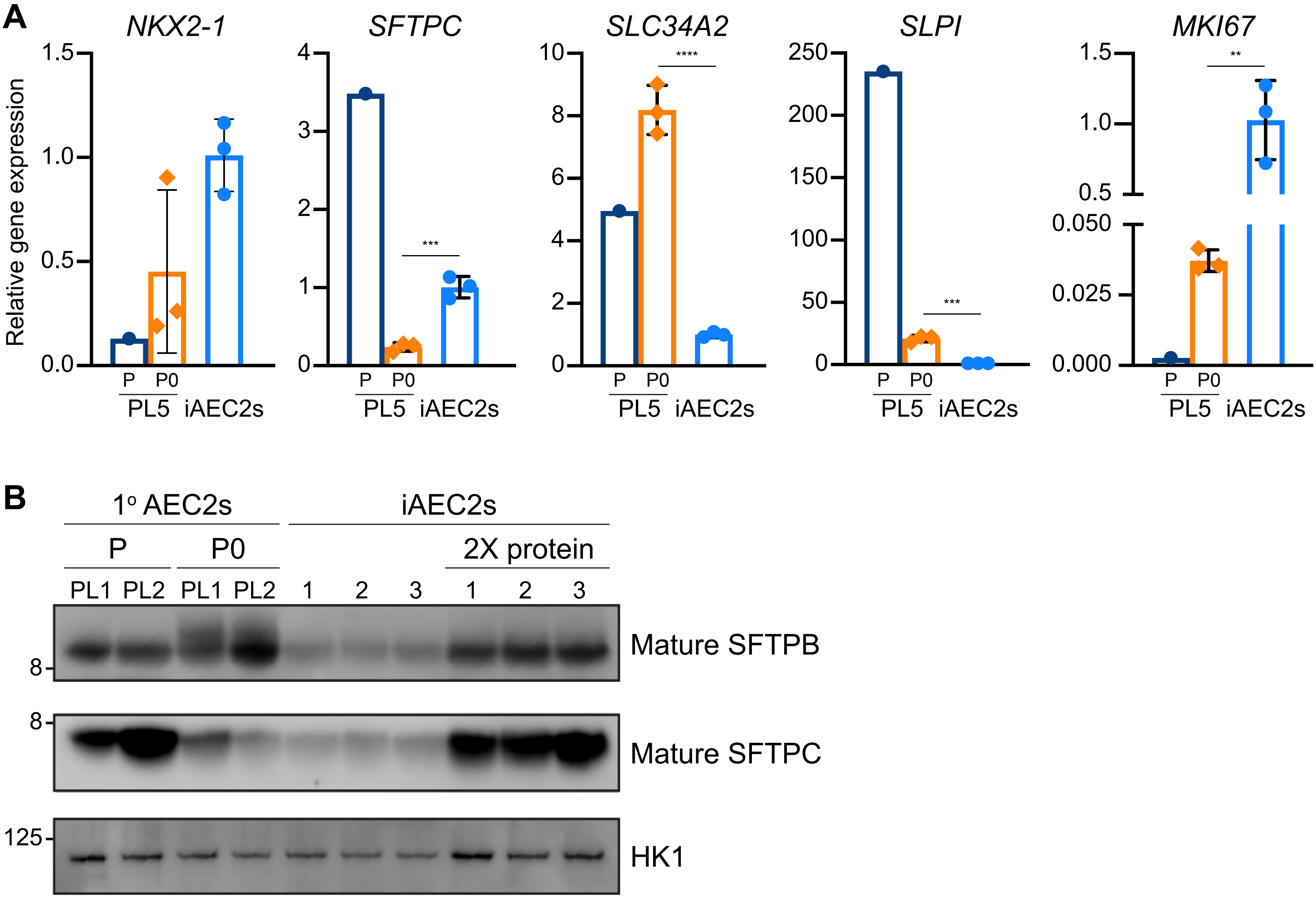

Supplemental Figure 1. Cultured AEC2s (1° and iAEC2s) acquire a less mature and more proliferative state compared to pre-culture 1° AEC2s, related to Figures 1 and 3.

(A) RT-qPCR showing fold change in gene expression compared to iAEC2s in pre-culture (P) and cultured (P0) 1° AEC2s from donor PL5, (n=3 experimental replicates). Mean ± SD is shown; **$p<0.01$, ***$p<0.001$, ****$p<0.0001$ by unpaired, two-tailed Student's t-test.

(B) Western blot analyses of 1° AEC2s and iAEC2 for surfactant protein expression. Cell lysates from pre-culture 1° AEC2s (P), matched cultured 1° AEC2s (P0), and iAEC2s (n=3) were subjected to SDS PAGE and western blotting as described in the Methods. To control for the presence of serum in 1° AEC2s samples, total protein loading of each lane was varied to normalize each sample to constant intracellular protein content based on GAPDH content. After transfer, mature SFTPB and SFTPC were detected using previously described immuno-reagents. Equal loading was confirmed by probing for the housekeeping gene Hexokinase 1 (HK1). The three lanes at the right represent duplicate iAEC2 lysates for which loading was increased as indicated to further demonstrate the presence of fully processed SFTPB and SFTPC isoforms.

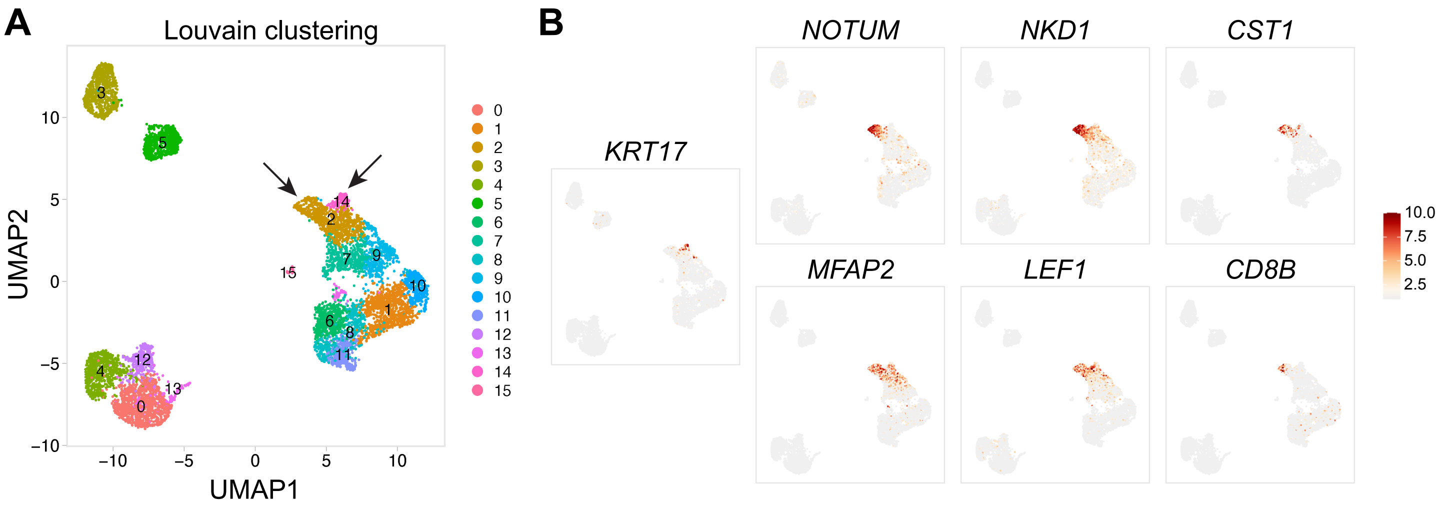

Supplemental Figure 2. Cluster 2 cells are closely related to cluster 14 cells and represent a subset of the transitional cell state, related to Figures 2, 7, and 8.
(A) Louvain clustering of cell transcriptomes showing 16 different clusters similar to Figure 2B. Arrows indicate clusters 2 and 14, respectively.
(B) Normalized gene expression overlaid on UMAP plots showing transcripts upregulated in cluster 2.

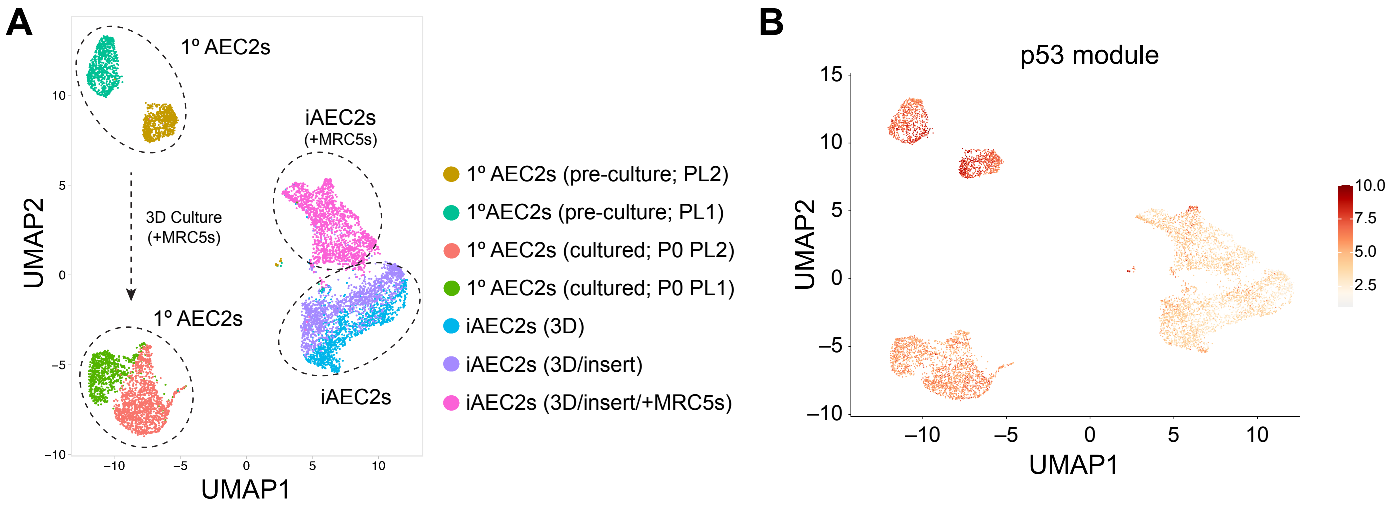

Supplemental Figure 3. Enriched expression of a p53 module score in cultured 1º AEC2s vs iAEC2s, related to Figures 4 and 5.

(A) Visualization of pre-culture 1º AEC2s, cultured 1º AEC2s, and iAEC2s scRNA-seq transcriptomes using Uniform Manifold Approximation Projection (UMAP) similar to Figure 2A.

(B) Normalized gene expression overlaid on UMAP plots for the p53 module generated using the Hallmark p53 pathway gene list.

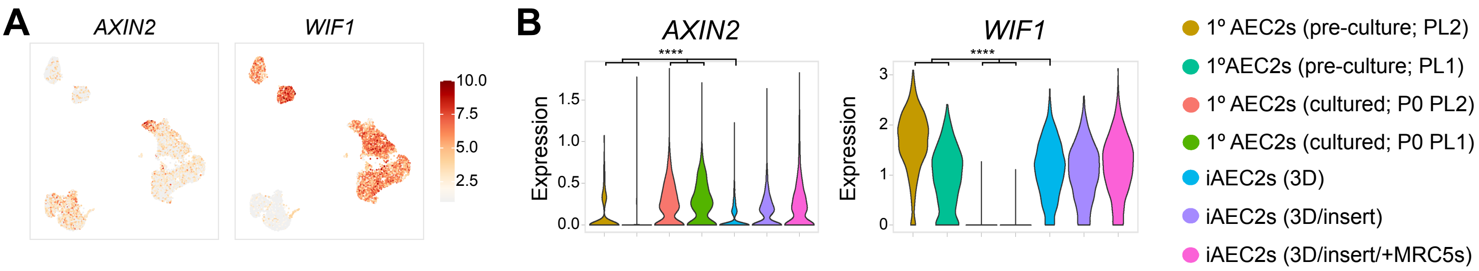

Supplemental Figure 4. Wnt-related genes profiled across samples, related to Figure 5.
(A) Normalized gene expression overlaid on UMAP plots for Wnt target genes AXIN2 and WIF1.
(B) Violin plots showing normalized expression for the Wnt target genes AXIN2 and WIF1 in pre-culture 1° AEC2s, cultured 1° AEC2s, and iAEC2s by scRNA-seq. ****p<0.0001 by one-way ANOVA with Bonferroni correction for multiple comparisons.

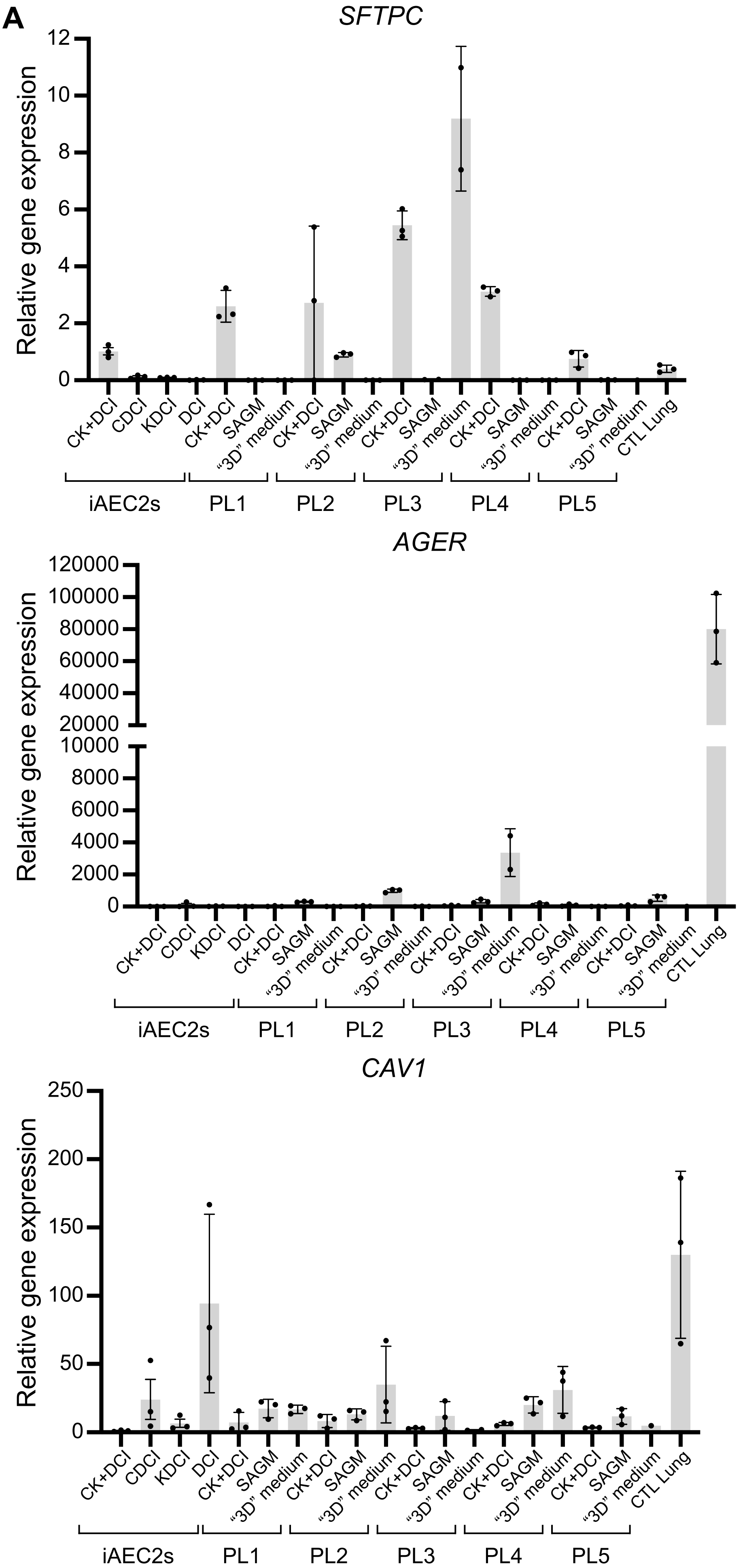

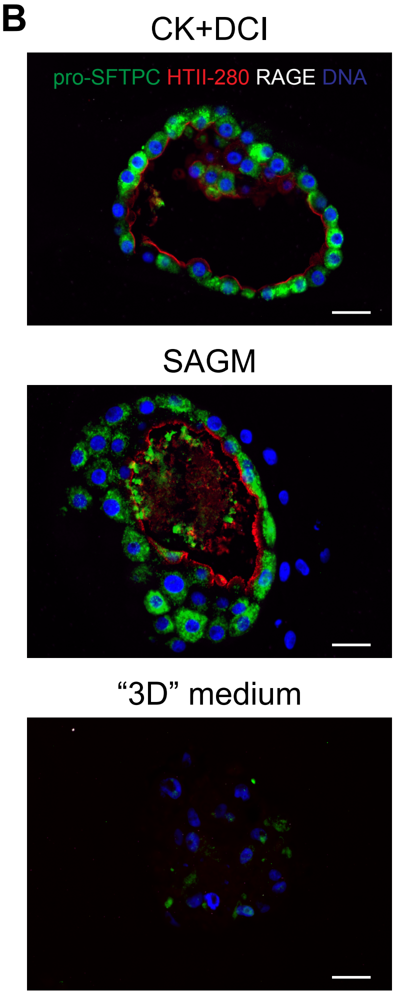

Supplemental Figure 5. Absence of expression of the AEC1 molecular phenotype in the evaluated AEC2 culture models, related to Figure 6.
(A) RT-qPCR showing fold change in gene expression compared to iAEC2s cultured in CK+DCI medium in iAEC2s cultured in CDCI, KDCI, or DCI medium as well as 1° AEC2s from 5 different donors (PL 1-5) cultured in CK+DCI medium, SAGM, or "3D" medium. Note the highest expression of the AEC2-specific transcript *SFTPC* in CK+DCI medium compared to other media across all samples and that no condition is associated with emergence of the full AEC1 phenotype in either 1° or iPSC-derived cultured AEC2s based on low or no expression of the AEC1-specific transcripts *AGER* and *CAV1* when compared to an adult human distal lung explant control (CTL Lung).
(B) Representative immunofluorescence microscopy of 1° AEC2 organoids (from donor PL2) cultured in the indicated media for pro-SFTPC (green), HTII-280 (red), RAGE (white), and DNA (Hoechst, blue). Scale bars: 25 μm. The lack of RAGE positive cells suggests that 1° AEC2s do not differentiate to AEC1s in these culture conditions.

Supplemental Table 1. Flow Cytometric Analysis Antibodies.

Antibody	Fluorochrome	Clone	Catalog Number	Manufacturer
EPCAM/CD326	PE-Cy7	9C4	324222	BioLegend
CD26	PE	BA5b	302705	BioLegend
CD31	PE	WM59	303106	BioLegend
CD45	PE	HI30	304012	BioLegend
CD47	PerCP-Cy5.5	CC2C6	323110	BioLegend
Mouse IgG1 isotype	PE	MOPC-21	400113	BioLegend
Mouse IgG1 isotype	PerCP-Cy5.5	MOPC-21	400149	BioLegend
HTII-280	N/A	N/A	TB-27AHT2-280	Terrace Biotechnology
Rat anti-mouse IgM	FITC	RMM-1	406505	BioLegend
NKX2-1/TTF1	N/A	EP15847	ab76013	Abcam

Supplemental Table 2. Antibodies.

**Immunofluorescence**				
**Antibody**	**Clonality**	**Dilution**	**Catalog Number**	**Manufacturer**
E-Cadherin/CDH1	Monoclonal	1:100	610181	BD Biosciences
HTII-280	Monoclonal	1:100	TB-27AHT2-280	Terrace Biotechnology
Pro-SFTPC	Polyclonal	1:1000	WRAB-9337	Seven Hills Bioreagents
RAGE	Polyclonal	1:100	AF1145	R&D Systems
Recombinant Alexa Fluor 647 Anti-Cytokeratin 8/KRT8 (clone EP1628Y)	Monoclonal	1:100	ab192468	Abcam
Recombinant Alexa Fluor 488 Anti-Cytokeratin 17/KRT17 (clone EP1623)	Monoclonal	1:100	ab185032	Abcam
Donkey anti-Goat IgG (H+L), Alexa Fluor Plus 594 conjugated	Polyclonal	1:500	A32758	Thermo Fisher Scientific
Donkey anti-Mouse IgG (H+L), Alexa Fluor Plus 594 conjugated	Polyclonal	1:500	A32744	Thermo Fisher Scientific
Donkey anti-Rabbit IgG (H+L), Alexa Fluor Plus 488 conjugated	Polyclonal	1:500	A32790	Thermo Fisher Scientific
ProLong Gold Antifade Mountant	N/A	N/A	P36934	Thermo Fisher Scientific
**Western Blot**				
SFTPB	Polyclonal	1:2500	PT3	(1)
Mature SFTPC	Polyclonal	1:1000	WRAB-76694	Seven Hills
Hexokinase 1	Polyclonal	1:2500	19662-1-AP	Proteintech
Goat Anti-Rabbit IgG (H + L)-HRP Conjugate		1:10000	170-6515	Bio-Rad

1. Beers MF, Bates SR, Fisher AB. Differential extraction for the rapid purification of bovine surfactant protein B. *Am J Physiol* 1992;262(6 Pt 1):L773-8.

**Supplemental Table 3. Oligonucleotides**		
TaqMan Gene Expression Assay Primer/Probe Sets		
AGER	Thermo Fisher Scientific	Hs00542584_g1
CAV1	Thermo Fisher Scientific	Hs00971716_m1
KRT8	Thermo Fisher Scientific	Hs01670053_m1
KRT17	Thermo Fisher Scientific	Hs00356958_m1
NKX2-1	Thermo Fisher Scientific	Hs00968940_m1
PDPN	Thermo Fisher Scientific	Hs00366766_m1
SFTPC	Thermo Fisher Scientific	Hs00161628_m1
SLC34A2	Thermo Fisher Scientific	Hs00197519_m1
18S	Thermo Fisher Scientific	4318839

SFTPB

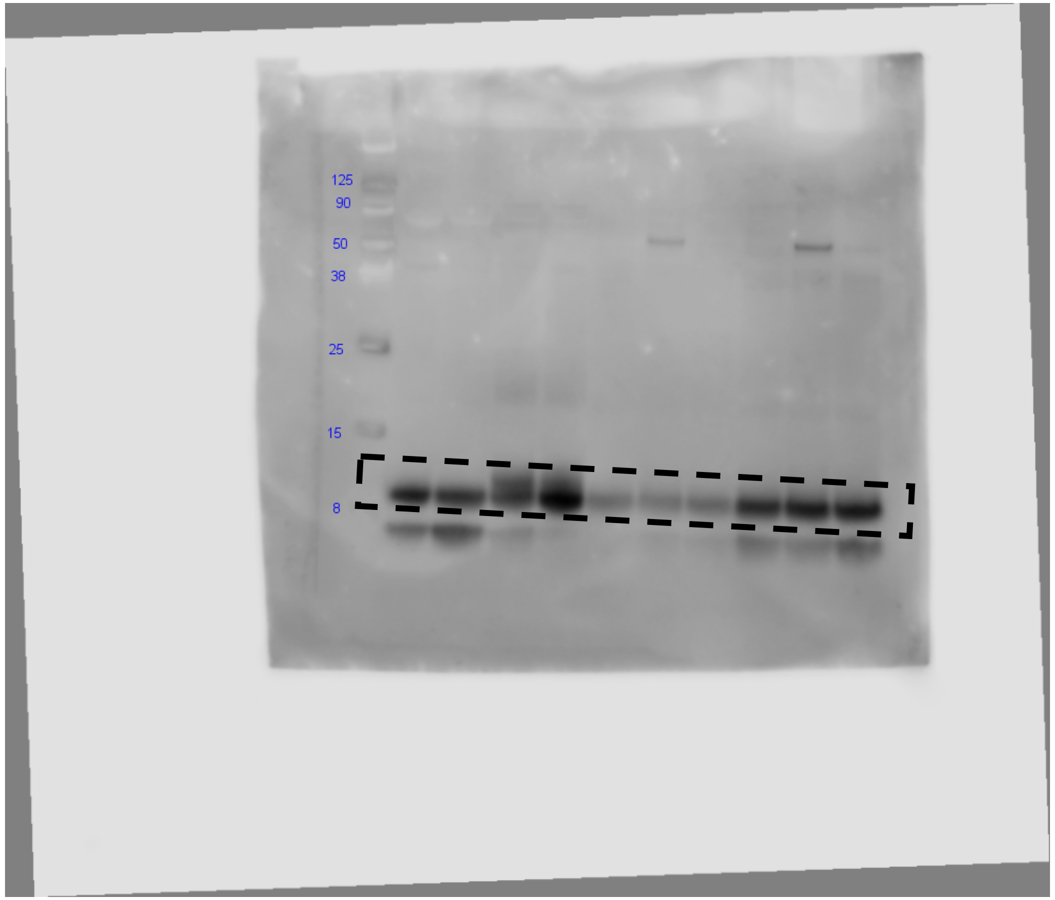

SFTPC

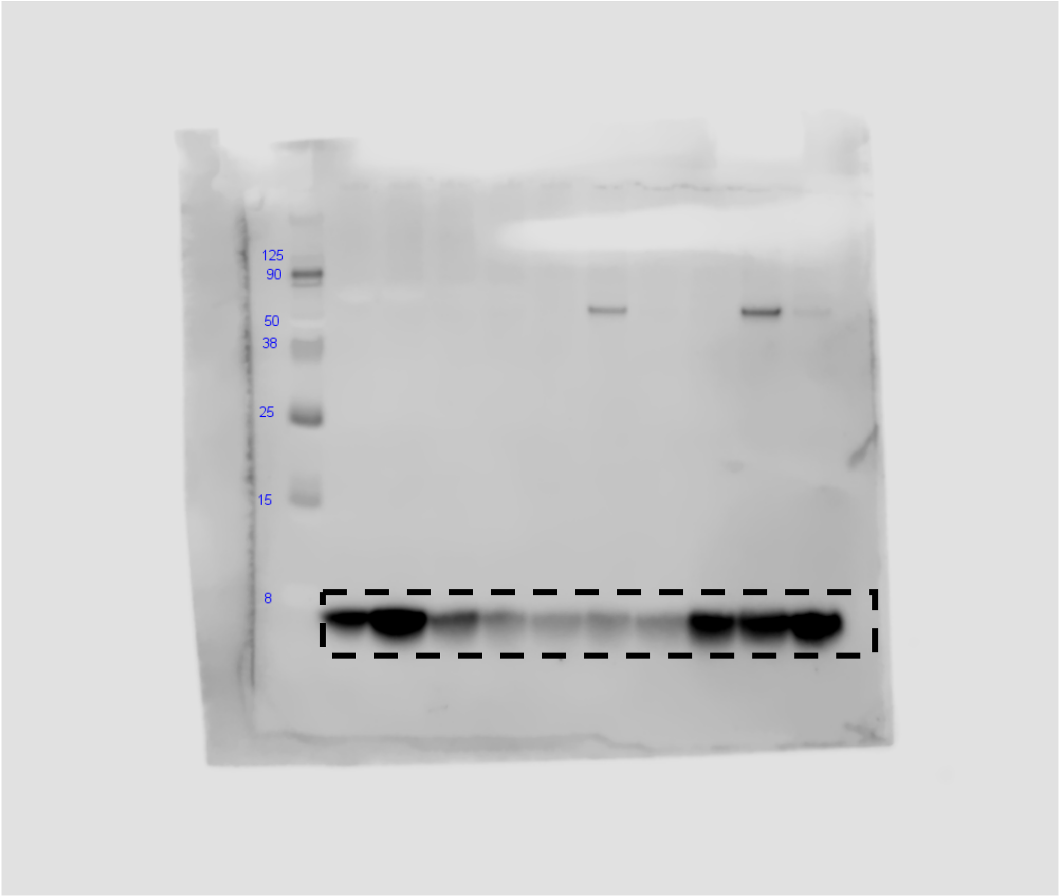

GAPDH

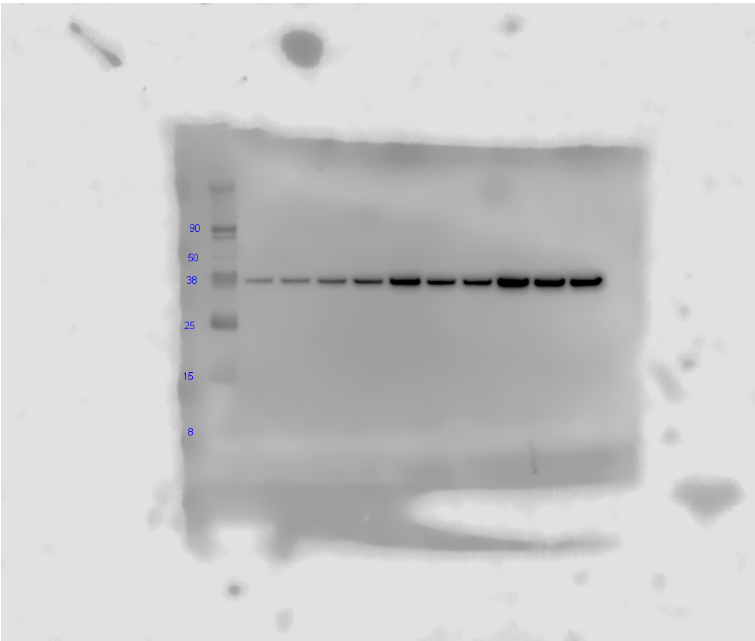

HK1

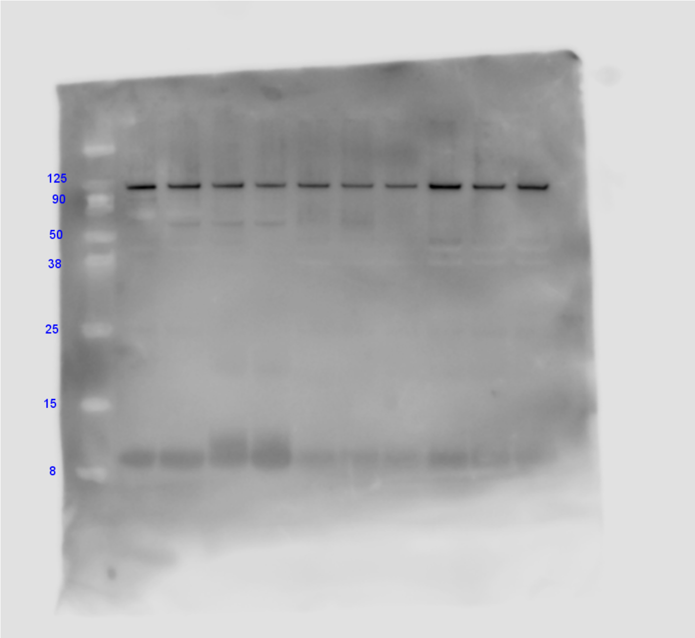

Total Protein

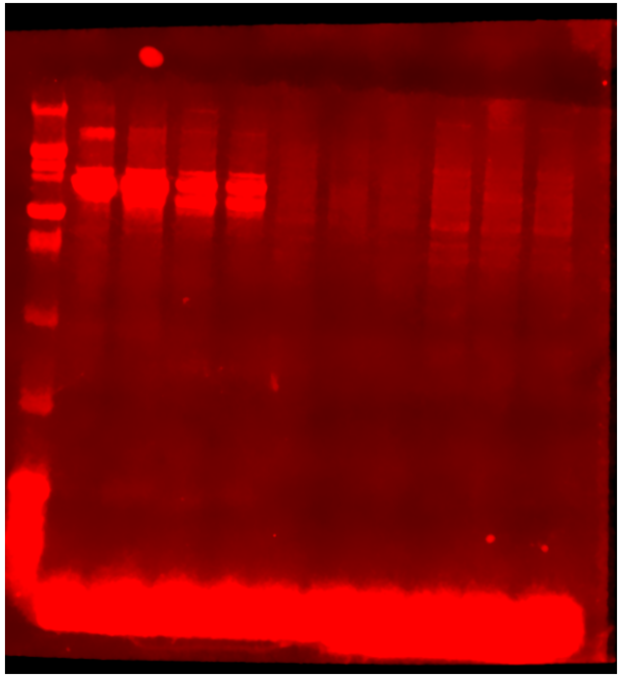

Supplement: Supplemental data [file jciinsight-8-158937-s055.pdf]
